# Atomic-Accuracy Prediction of Protein Loop Structures through an RNA-Inspired Ansatz

**DOI:** 10.1371/journal.pone.0074830

**Published:** 2013-10-21

**Authors:** Rhiju Das

**Affiliations:** Departments of Biochemistry and Physics, Stanford University, Stanford, California, United States of America; University Of Oxford, United Kingdom

## Abstract

Consistently predicting biopolymer structure at atomic resolution from sequence alone remains a difficult problem, even for small sub-segments of large proteins. Such loop prediction challenges, which arise frequently in comparative modeling and protein design, can become intractable as loop lengths exceed 10 residues and if surrounding side-chain conformations are erased. Current approaches, such as the protein local optimization protocol or kinematic inversion closure (KIC) Monte Carlo, involve stages that coarse-grain proteins, simplifying modeling but precluding a systematic search of all-atom configurations. This article introduces an alternative modeling strategy based on a ‘stepwise ansatz’, recently developed for RNA modeling, which posits that any realistic all-atom molecular conformation can be built up by residue-by-residue stepwise enumeration. When harnessed to a dynamic-programming-like recursion in the Rosetta framework, the resulting stepwise assembly (SWA) protocol enables enumerative sampling of a 12 residue loop at a significant but achievable cost of thousands of CPU-hours. In a previously established benchmark, SWA recovers crystallographic conformations with sub-Angstrom accuracy for 19 of 20 loops, compared to 14 of 20 by KIC modeling with a comparable expenditure of computational power. Furthermore, SWA gives high accuracy results on an additional set of 15 loops highlighted in the biological literature for their irregularity or unusual length. Successes include *cis*-Pro touch turns, loops that pass through tunnels of other side-chains, and loops of lengths up to 24 residues. Remaining problem cases are traced to inaccuracies in the Rosetta all-atom energy function. In five additional blind tests, SWA achieves sub-Angstrom accuracy models, including the first such success in a protein/RNA binding interface, the YbxF/kink-turn interaction in the fourth ‘RNA-puzzle’ competition. These results establish all-atom enumeration as an unusually systematic approach to *ab initio* protein structure modeling that can leverage high performance computing and physically realistic energy functions to more consistently achieve atomic accuracy.

## Introduction

Atomic-resolution prediction of protein three-dimensional structure is a biophysical problem with fundamental implications for the structure determination and rational engineering of complex biological systems [1]–[4]. Recent years have seen major successes in modeling protein structure through the *in silico* optimization of all-atom energy functions [2]–[4]. However, as assessed in blind trials, these computational algorithms achieve atomic accuracy only in favorable cases [5]–[7] or when guided by experimental data [8]–[10], even with the application of new kinds of specialized supercomputers [11]. Even relatively short sequences, such as loops involved in catalysis or in binding of drugs or macromolecule partners, present a massive number of possible conformations that cannot be exhaustively searched [1], [12]. Most available methods thus make use of coarse-grained search phases using knowledge-based potentials or approximate filters to reduce the number of energy minima that need to be searched [2], [4]–[6], [8], [10], [12].

Recently, a conceptually distinct approach to modeling macromolecule structure has arisen from efforts to predict complex RNA structures in all-atom detail [13]–[16]. A working hypothesis, called a ‘stepwise ansatz’, posits that native biopolymer structures can be built through the systematic step-by-step addition of one residue at a time. When integrated via a dynamic-programming recursion, this ansatz permits the enumeration of a physically realistic subspace of molecular conformations at all-atom resolution, and was implemented as a stepwise assembly (SWA) protocol in the Rosetta framework. In a comprehensive benchmark, this *ab initio* method consistently eliminated conformational sampling bottlenecks and solved RNA loops and motifs at high resolution. The method has furthermore been successful in blind tests [13], [14], but in all cases has required the expenditure of significant computational power (thousands of CPU-hours). Fortunately, when coupled to limited experimental data, SWA can be accelerated and is enabling the determination of difficult NMR structures from limited RNA chemical shift data ([17], [18]; Sripakdeevong, P. and RD, submitted) and the automated correction of errors in fitting RNA coordinates into crystallographic density maps (the ERRASER method [15], [18], [19]).

Given its advantages over prior RNA modeling approaches and its assurance of complete sampling, stepwise assembly also holds promise for difficult problems in protein structure prediction. This study presents the first application of SWA to proteins, focusing on loop modeling. Protein loop modeling problems arise frequently in comparative modeling, designing new proteins, and solving or refining protein folds with limited crystallographic or NMR data, including weakly populated (‘invisible’) states [20]–[24]. Even the simplest ‘toy puzzle’ of re-building a loop excised from a crystallographic structure has remained difficult to consistently solve when the side-chains inside and outside the loop are erased, mimicking a realistic prediction scenario. Such a problem involves searching a large number of degrees of freedom – dozens of backbone torsions and hundreds of side-chain torsions – to achieve a precise ‘lock-and-key’ fit between the loop and the surrounding protein [21], [22]. Despite important methodological advances in recent years, inefficient conformational sampling has continued to be a general bottleneck in protein loop modeling [21], [22], [24].

**Figure 1:**
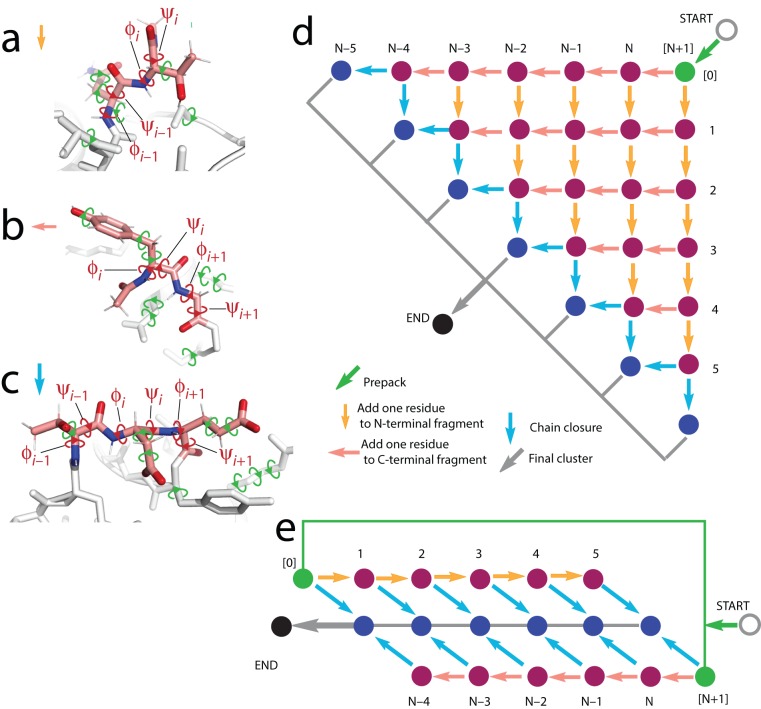
Schematics of stepwise assembly calculation. (**a**–**c**) Degrees of freedom sampled by residue-level enumeration (red torsions in backbone, labeled) and by side-chain combinatorial optimization (green torsions) for addition to N-terminal fragment (**a**), addition to C-terminal fragment (**b**), and chain-closure step (**c**). In (a) and (b), note presence of methylamide and acetyl ‘caps’, respectively, to model peptide connection to next residue. (**d**) Directed acyclic graph (DAG) outlining overall calculation. Movements leftward or downward in the graph indicate building on loop N-terminal fragment and C-terminal fragment, respectively. Each filled circle represents a stage (*i*, *j*) at which models are clustered. The diagram is for a loop with *N* = 6 residues. Chain closure steps (cyan arrows) for models with one-residue gap between N- and C- terminal fragments are shown; for clarity, steps that close two- or three- residue gaps are not shown. (**e**) Simplified DAG in which fragments are built from N-terminal end without concomitant growth in C-terminal end, or vice versa, followed by chain closure. This calculation takes O (*N*) computational expense, compared to O(*N* ^2^) expense of the full DAG in (a).

To address the conformational sampling bottleneck, this article describes the import of stepwise assembly from RNA modeling to protein loop structure prediction in the Rosetta framework (Figure 1). The resulting SWA method is tested in a benchmark of thirty-five protein loops, including numerous cases that have been challenges for prior approaches. The benchmark includes loops with conformations ranging from corkscrews to hairpins, complex motifs such as *cis*-Pro touch turns, loops that thread through tunnels formed by the surrounding protein, and segments of unprecedented length. These cases are solved *ab initio* by SWA with sub-Angstrom accuracy, albeit at the expense of thousands of CPU-hours per loop. Additional atomic-accuracy results from five blind predictions, including a comparative modeling problem for a protein/RNA complex, give further support to the stepwise ansatz and its Rosetta SWA implementation. Analogous to recent successes of RNA modeling in structural biology [13]–[16], [19], all-atom conformational enumeration may be useful for systematically dissecting protein structure and dynamics in practical scenarios with limited experimental data.

## Results

### Difficulty of loop structure prediction with atomic accuracy

**Figure 2:**
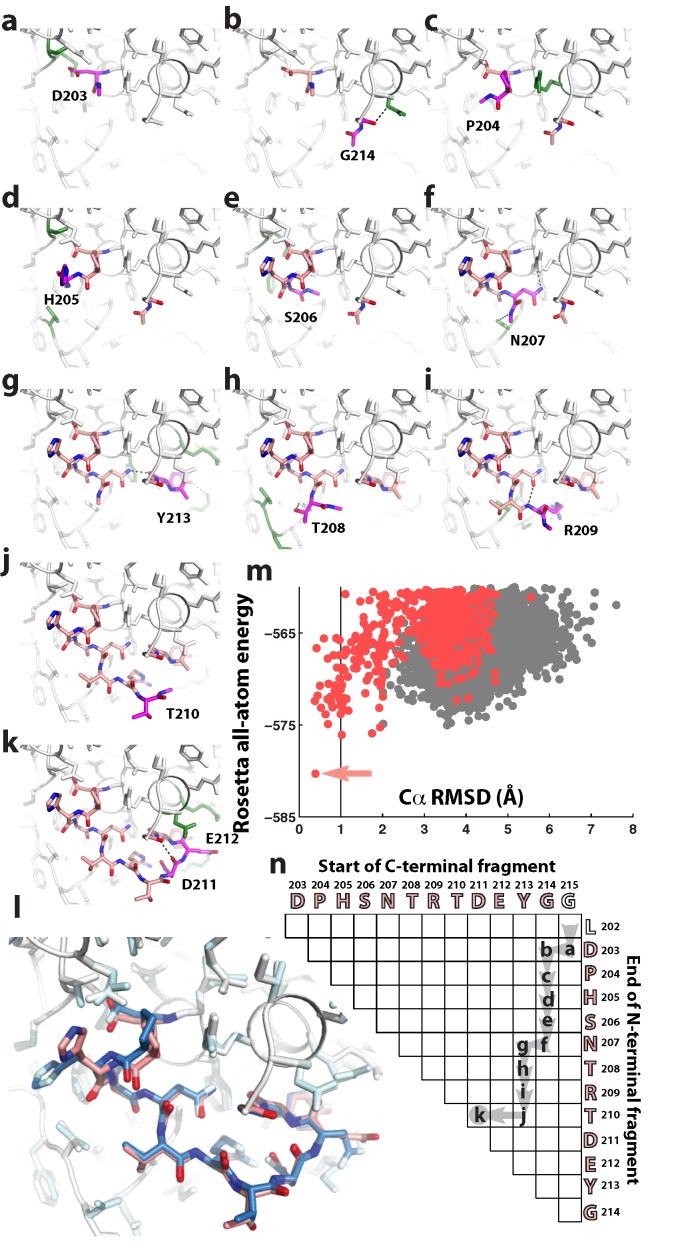
Stepwise assembly applied to a challenging loop prediction problem. (**a**–**k**) Stepwise assembly (SWA) of residues 203–214 from PDB ID *1oyc*. In each panel, the added residue (carbon atoms in magenta), previously built loop residues (pink), surrounding side-chains that interact with the new residue (green), and newly formed hydrogen bonds (dashed lines) are highlighted. (**l**) Final loop (pink) from this build-up path (a–k) agrees with the crystallographic loop (blue) with atomic accuracy (Cα RMSD 0.39 Å). Surrounding side-chains are shown in white (SWA model) and pale cyan (crystallographic model). (**m**) Energy vs. RMSD of all SWA models (red), generated by recursively following all build-up paths, compared to models from Rosetta kinematic closure Monte Carlo (gray). Arrow marks the lowest energy model discovered; this is the conformation in (k) and (l). (**n**) Dynamic-programming-style matrix highlighting the residue-by-residue build-up in path (a–k).

Protein loops provide well-defined problems for structure prediction, as exemplified in Figure 2 by a 12-residue segment of a flavoenzyme (sequence DPHSNTRTDEYG; residues 203–214 in PDB entry *1oyc*), an unsolved case in recent studies [21], [22]. Compared to regions of regular secondary structure, protein loops contain similar numbers of hydrogen bonds (1.1 per residue, on average; see *N*_HB_ in Table 1), but contain few ‘regular’ α-helix-like or β-sheet-like backbone-backbone hydrogen bonds (only two in the *1oyc* case). Furthermore, loops make few interactions with the non-polar core, arguably the best modeled region of protein structures [2], [25], [26], and present few non-polar side-chains. For example, nearly all of the *1oyc* loop residues (Table 1) have charged or polar side-chains, which require atomically precise positioning to form hydrogen bonds; and the other residues are glycine and proline, which each have unique conformational properties [27].

**Table 1:** Loop sequences and sources for all test cases.

PDB	Loop	N	N_contact_ a		N_out_ b		N_SC_ c	N_HB_ d		Loop sequence			Source
**PLOP/Rosetta benchmark**													
1a8d	155–166	12		4.8		2.9			1.9		1.4	DLPDKFNAYLAN	[21], [22]
1arb	182–193	12		5.2		4.2			1.3		2.1	WQPSGGVTEPGS	[21], [22]
1bhe	121–132	12		3.4		2.4			1.1		1.2	GQGGVKLQDKKV	[21], [22]
1bn8	298–309	12		3.8		2.5			0.9		0.8	STSSSSYPFSYA	[21], [22]
1c5e	82–93	12		2.9		2.4			1.1		0.9	YEDVLWPEAASD	[21], [22]
1cb0	33–44	12		3.8		2.8			1.4		1.4	YVDTPFGKPSDA	[21], [22]
1cnv	188–199	12		4.3		3.0			1.3		1.2	FYNDRSCQYSTG	[21], [22]
1cs6	145–156	12		2.8		2.7			1.4		1.1	NEFPNFIPADGR	[21], [22]
1dqz	209–220	12		3.1		2.6			0.8		0.8	CGNGTPSDLGGD	[21], [22]
1exm	291–302	12		4.1		2.6			1.4		1.3	RGVSREEVERGQ	[21], [22]
1f46	64–75	12		2.6		2.2			1.1		0.6	MVKPGTFDPEMK	[21], [22]
1i7p	63–74	12		3.9		3.1			1.1		1.2	LPSPQHILGLPI	[21], [22]
1m3s^e^	68–79	12		2.2		1.8			0.7		1.2	VGEILTPPLAEG	[21], [22]
1ms9	529–540	12		2.5		2.5			0.8		0.8	GSTPVTPTGSWE	[21], [22]
1my7	254–265	12		2.9		2.8			0.8		0.5	TPPYADPSLQAP	[21], [22]
1oth	69–80	12		2.7		2.3			0.8		1.0	QKGEYLPLLQGK	[21], [22]
1oyc	203–214	12		4.9		3.6			1.9		1.9	DPHSNTRTDEYG	[21], [22]
1qlw	31–42	12		2.9		1.4			0.8		1.0	ETLSLSPKYDAH	[21], [22]
1t1d	127–138	12		3.1		2.4			0.7		1.1	SGGRLRRPVNVP	[21], [22]
2pia	30–41	12		2.6		2.1			0.6		0.9	DPQGAPLPPFEA	[21], [22]
**Difficult cases**													
1alc	34–41	8		5.2		4.5			1.1		1.2	SGYDTQAI	[21], [58]
1msp	54–62	9		3.4		3.4			0.9		1.0	SVDPPCGVL	[35]
1w7z	40952	12		1.9		1.9			0.8		0.6	CPRILIRCKQDS	[33], [59] f
2tgi	48–59	12		3.6		2.9			1.4		1.8	CPYLWSSDTQHS	[22], [60]
1ppn	175–186	12		4.9		3.1			1.9		1.2	NSWGTGWGENGY	[36], [61] f
1bni	75–86	12		4.2		2.7			1.4		1.6	DINYTSGFRNSD	[62], [63] f
2ci2	53–64	12		1.8		1.8			0.6		0.7	VGTIVTMEYRID	[33], [64], [65]
1udg	152–163	12		3.8		2.1			1.2		1.5	VKRGAAASHSRI	[66] f
1arp	290–307	18		3.1		2.8			1.1		0.8	LTDCSDVIPSAVSNNAAP	[34]
1huw	47–64	18		2.4		1.9			1.1		1.0	NPQTSLCPSESIPTPSNK	[60]
1rhd^f^	136–153	18		2.3		2.3			0.8		0.7	EGHPVTSEPSRPEPAIFK	[60]
7cat	290–309	20		3.8		2.4			1.7		0.9	IFPFNPFDLTKVWPHGDYPL	[60]
1thg	309–332	24		4.8		3.0			1.3		1.5	LFGLLPQFLGFGPRPDGNIIPDAA	[22], [60]
1c5e^f^	70–93	24		4.2		3.6			1.1		1.1	TTLTFYKSGTFRYEDVLWPEAASD	[21], [22]
1rhd	136–164	29		2.8		2.6			0.8		0.7	EGHPVTSEPSRPEPAIFKATLNRSLLKTY	[60]
**Blind tests**													
Test A	22–33	12		1.0		0.7			0.2		0.3	_g	Blind^g^
Test B	57–68	12		2.4		1.8			0.4		1.2	_g	Blind^g^
Test C	92–103	12		3.6		1.8			1.1		2.0	_g	Blind^g^
Test D	210–220	11		2.7		1.6			0.4		1.2	_g	Blind^g^
3v7e	534–545	12		3.8		2.2			0.8		1.8	AKDADPILTSSV	Blind [39]
Mean		13.2		3.3		2.5			1.0		1.1		
Median		12.0		3.1		2.5			1.1		1.1		

For statistics N_contact_, N_out_, N_SC_, and N_HB_, residues with sequence positions within two residues of each loop residue were excluded from the calculation; only non-hydrogen atoms were considered.

a Average number of residues that make at least one atom-atom contact (distance <4.0 Å) with each loop residue.

b Avg. number of residues outside the loop that make an atom-atom contact (dist. <4.0 Å) with each loop residue.

c Avg. number of residues that make an atom-atom contact (dist. <4.0 Å) to a loop residue involving an atom requiring side-chain placement (not N, C, Cα, Cβ, O).

d Avg. number of hydrogen bonds per residue, defined as donor/acceptor pairs with distance less than 3.2 Å.

e Test included two crystallographic neighbors that interact with loop [21], [22].

f Loop with irregular structure that remains rigid upon binding to inhibitors or protein partners; see cited references.

g Unpublished 275-residue protein crystal structure from W. Weis and colleagues (see Figs. 5A–D).

These factors have made the *1oyc* test case difficult for state-of-the-art methods, including the Protein Loop Optimization Protocol (PLOP), which uses backbone-only hierarchical loop build-up followed by molecular mechanics force field refinement [20], [21]; fragment-assembly modeling with all-atom refinement in the Rosetta framework [22], [24]; and a more powerful Rosetta algorithm based on Monte Carlo sampling with exact kinematic loop closure (KIC) [22]. To further test the limits of state-of-the-art methods, Rosetta KIC modeling has been repeated herein with several enhancements, including generation of 16,800 models (greater than 16-fold times the computational expenditure of prior work [22]), *cis* proline sampling, and more stringent chain closure. Even in these more extensive calculations (Table 1), the best of five lowest energy cluster centers for the *1oyc* loop achieved a Cα RMSD accuracy of 3.03 Å, significantly worse than RMSD values less than 1.0 Å associated with atomic accuracy solutions [20]–[22]. Indeed, none of the KIC models attained better than 1.68 Å RMSD to the crystallographic loop. Nevertheless, as shown below, near-native loop configurations with substantially lower all-atom Rosetta energies exist for the loop. These results illustrate the difficulty of high-resolution protein loop structure prediction, even with the application of large amounts of computational power.

### Importing a stepwise ansatz from RNA modeling

Most current loop modeling approaches that seek atomic resolution share a seemingly necessary working approximation: an initial search phase using a reduced representation with simplified or no side-chain atoms. Such coarse search phases avoid the complexity and large number of local minima inherent to all-atom representations, but fail to capture non-polar packing interactions and hydrogen bonds involving side-chain atoms, which are pervasive (see *N*_SC_ and *N*_HB_ in Table 1). A strategy that ensures sufficient sampling, or even enumeration, of such interactions in all-atom detail would appear desirable. However, even if constrained to fixed bond lengths and angles, planar *trans*-peptide bonds (ω = 180°), crystallographically observed (φ,ψ) combinations, and closed loop geometries, sampling each amino acid at sub-Angstrom resolution requires at least tens of backbone conformers (see SI Methods), resulting in at least 10^12^ backbone torsional combinations for a 12-residue loop. Subsequent side-chain optimization on these backbones would require tens of millions of CPU-hours [28]. Furthermore, the combinatorial space becomes exponentially larger with increasing loop length.

Recent work in three-dimensional RNA modeling [13] has suggested a distinct strategy for conformational enumeration, based on the following working hypothesis, or ansatz: the native conformation of a loop can be built up progressively in small steps such that each intermediate partial conformation is itself a well-packed, low-energy configuration (Figure 1). As implemented herein, the steps involve adding a single residue to either a fragment growing from the N-terminus or the C-terminus of the loop (Figs. 1A and 1B) and bridging the two fragments when they arrive within three residues of each other (Fig. 1C). Each step involves three sub-steps: enumeration of backbone torsions for the new residue and any sequence-adjacent loop residues; optimization of the new and nearby side-chains; and minimization of the entire loop and surrounding protein side-chains (see Methods for detailed descriptions of residue capping, torsions sampled, chain closure algorithm, and example Rosetta command-lines). An ensemble of 400 models is retained and typically includes models within 5 *k*_B_ *T* of the lowest energy model at each stage, mimicking a thermal ensemble (here, a Rosetta energy unit is taken to be approximately one *k*_B_ *T* [29]). Each step is enumerative and deterministic except the local side-chain optimization. While this side-chain optimization problem can be solved exactly [30], [31], SWA relies on Rosetta's stochastic one-at-a-time sampling [28] for speed; the resulting side-chain search appears near-optimal, as independent trials of the entire stepwise calculation give lowest-energy final models with similar conformations (<0.5 Å RMSD) and energies (within 1–2 *k*_B_ *T*). This protocol has been coded into the Rosetta framework as a stepwise assembly (SWA) algorithm.

As an example, Figs. 2A–K illustrate the step-by-step building of a near-native *1oyc* loop. For the first five residues (N-terminal 203–206 & C-terminal 214; Figs. 2A–E), conformations within 0.6 Å of the crystallographic loop are observed in the ensembles of 400 lowest energy models for the growing chain, although not as the very lowest energy model (see Fig. S1 for full energy vs. RMSD plots). The features that stabilize these near-native conformations all involve side-chains: a side-chain hydrogen bond from D203, a backbone-to-side-chain hydrogen bond from G214, non-polar packing by the P204 prolyl ring, separate packing interactions by H205, and again a side-chain hydrogen bond from S206. In prior approaches, initial search phases that omit or coarse-grain side-chains for computational simplicity would necessarily miss these key, atomic-level details. The SWA modeling of the *1oyc* loop then continues through additional rebuilding steps from the N- and C- termini and chain closure across residues D211-E212 (Figs. 2F–K). The final lowest energy model for the full loop achieves a Cα RMSD of 0.39 Å, with all backbone and side-chain hydrogen bonds recovered with atomic accuracy (Fig. 2L). Reaching this final lowest energy solution requires intermediate structures that are not the lowest energy at their intermediate build-up stages (Fig. S1), underscoring the need for keeping a full ensemble of models during the build-up procedure.

**Figure 3:**
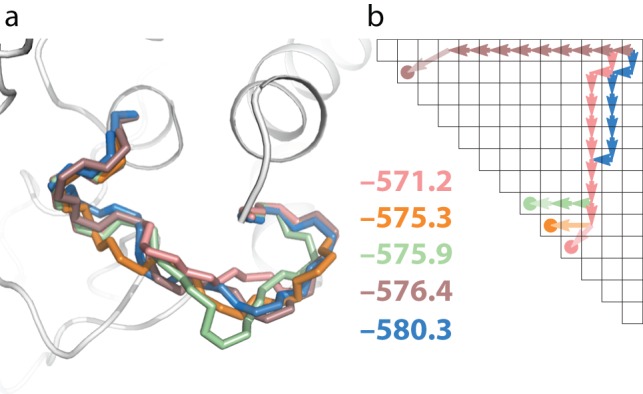
Effect of build-up path on loop conformations. (**a**) Five low energy conformations, and (**b**) corresponding build-up paths and Rosetta all-atom energies (numerical values given in Rosetta units, approximately 1 *k*_B_ *T*) from the *1oyc* test case of Figure 1. Different build up paths can give similar configurations (compare brown and blue loops). Similar but distinct paths can give substantially different configurations (compare green, orange, and pink loops).

The preceding description outlined one potential residue-by-residue build-up path, but it is not known *a priori* which path, if any, will lead to the native loop. It is therefore critical to search through all possible build-up paths. This task is simplified by the observation that each path shares most of its sub-paths with other paths. The full build-up can thus be accomplished through a dynamic-programming-style recursion similar to methods in modeling RNA secondary structure [32] and, more recently, tertiary structure [13]. Briefly, each intermediate in the SWA build-up can be indexed by the ends of the N-terminal and C-terminal fragment of the loop; the ensembles to be modeled can thus be laid out schematically in a two-dimensional matrix (Fig. 1D). Members of each ensemble are computed by applying the build step to all relevant models in a previous ensemble (arrows to immediately neighboring matrix elements in Fig. 1D), followed by clustering of the resulting ensemble and retaining the lowest 400 models. The resulting number of build steps grows quadratically, rather than exponentially, in the number of residues in the loop and can be carried out with approximately one day of computation on a 200-CPU cluster (5,000 CPU-hours). [A simpler and faster recursion (Fig. 1E) that assumes limited interaction between C- and N- terminal fragments of the loop has also been implemented and tested (see Methods and Table 2), and can be carried out in advance of the full recursion.] The importance of search path in sampling diverse loop conformations is illustrated by the large differences in models with comparably low energy achieved by following different paths, as shown in Fig. 3. Nevertheless, for the *1oyc* case, the high-accuracy 0.39 Å conformation (Fig. 2L) remains the lowest energy loop after following all rebuild paths (Figs. 2M and 3B). This SWA model's energy is substantially lower than any conformation produced by Rosetta KIC Monte Carlo modeling (Fig. 2M).

**Table 2:** Accuracy achieved on 40 loop modeling cases.

Target	Length	Cα RMSD to crystallographic loop (Å)					
		Lowest RMSD model		Best of five models (rank)		Lowest energy model	
		KIC	SWA	KIC	SWA	KIC	SWA
PLOP/Rosetta benchmark							
1a8d^a^	12	0.71	0.33	0.71 (1)	0.42 (1)	0.71	0.42
1arb^a^	12	0.59	0.48	1.54 (1)	0.48 (1)	1.54	0.48
1bhe	12	0.50	0.30	0.61 (1)	0.30 (1)	0.61	0.30
1bn8	12	0.51	0.33	0.92 (1)	0.63 (2)	0.92	1.27
1c5e^b^	12	0.34	0.36	0.36 (1)	0.44 (2)	0.36	1.25
1cb0^a^	12	0.46	0.32	0.56 (1)	0.64 (1)	0.56	0.64
1cnv	12	0.84	1.22	1.59 (1)	1.59 (1)	1.59	1.59
1cs6^a^	12	0.93	0.66	1.94 (4)	0.79 (1)	2.87	0.79
1dqz^a^	12	0.52	0.38	0.76 (1)	0.48 (1)	0.76	0.48
1exm^a^	12	0.65	0.48	0.98 (1)	0.62 (1)	0.98	0.62
1f46	12	0.50	0.38	0.57 (1)	0.38 (1)	0.57	0.38
1i7p	12	0.39	0.39	0.49 (2)	0.43 (3)	2.83	1.61
1m3s^a^	12	0.27	0.27	0.36 (1)	0.27 (2)	0.36	3.24
1ms9	12	0.24	0.34	0.39 (1)	0.34 (1)	0.39	0.34
1my7	12	0.35	0.34	0.75 (1)	0.51 (1)	0.75	0.51
1oth^a^	12	0.31	0.43	0.39 (1)	0.71 (1)	0.39	0.71
1oyc	12	1.68	0.38	3.03 (2)	0.39 (1)	4.53	0.39
1qlw	12	1.00	0.45	1.24 (1)	0.66 (3)	1.24	4.98
1t1d	12	0.45	0.31	0.90 (1)	0.41 (1)	0.90	0.41
2pia^a^	12	0.67	0.55	1.10 (1)	0.83 (3)	1.10	1.06
Difficult cases							
1alc^a^	8	0.17	0.29	0.25 (1)	0.58 (2)	0.25	0.7
1msp^a^	9	0.27	0.52	0.55 (1)	0.73 (1)	0.55	0.73
1w7z	12	0.35	0.40	0.80 (1)	0.79 (1)	0.80	0.79
2tgi	12	0.70	0.53	1.57 (2)	0.53 (3)	2.73	2.87
1ppn	12	0.36	0.44	0.70 (1)	0.89 (1)	0.70	0.89
1bni	12	0.73	0.74	1.76 (2)	1.10 (4)	2.79	1.12
2ci2	12	0.69	0.82	2.35 (2)	3.44 (5)	2.73	4.50
1udg	12	0.48	0.53	0.94 (4)	2.43 (3)	1.92	3.29
1arp^a^	18	0.83	0.81	0.94 (1)	1.60 (1)	0.94	1.60
1huw^a^	18	0.67	1.16	0.67 (3)	1.35 (1)	1.92	1.35
1rhda,b	18	0.87	0.74	0.87 (1)	0.82 (2)	0.87	1.66
7cat^a^	20	4.02	7.18	6.86 (5)	7.69 (1)	7.65	7.69
1thg^a^	24	3.90	0.74	5.44 (2)	0.80 (1)	6.88	0.80
1c5ea,b	24	2.02	0.28	2.02 (2)	0.41 (1)	7.47	0.41
1rhda,b	29	4.67	3.57	11.28 (4)	12.20 (5)	16.43	17.63
Blind tests							
Test A	12	0.46	0.65	0.74 (1)	0.91 (3)	0.74	2.24
Test B	12	0.52	0.49	1.03 (1)	0.91 (1)	1.03	0.91
Test C	12	0.58	0.38	0.78 (2)	0.54 (1)	1.44	0.54
Test D	11	0.23	0.29	0.38 (1)	0.52 (1)	0.38	0.52
3v7e^a^	12	0.51	0.42	0.82 (2)	0.53 (1)	1.62	0.53
Mean	13.5	0.87	0.74	1.50	1.25	2.09	**1.81**
Median	12	0.80	0.44	0.84	0.64	0.96	**0.80**
RMSD <1.0 Å		34/40	**36/40**	26/40	32/40	22/40	23/40

a SWA runs carried out with simplified O(*N*) calculation scheme; see methods.

b Longer and shorter variants of loops were modeled separately; see Table 1.

The SWA method posits that the experimentally observed conformation of a protein loop is part of a low energy subspace that can be enumerated through the stepwise, locally optimal building of small subsegments. This working hypothesis – the stepwise ansatz [13] – appears feasible for native macromolecule conformations, in which nearly every residue makes precise, atomic-level interactions with other residues (see, e.g., Figs. 2A–K, and *N*_contact_, *N*_out_, *N*_SC_, and *N*_HB_ entries in Table 1). As with prior RNA methods [13], however, general confirmation of the ansatz requires extensive empirical tests on a wide range of protein loop structures, described next.

### Benchmarking the SWA algorithm

To test the stepwise ansatz, its Rosetta stepwise assembly (SWA) implementation was used to carry out structure prediction on a benchmark set of thirty-five protein loops. Twenty of these cases were 12-residue loops used previously to test PLOP and Rosetta approaches [21], [22]. Fifteen additional cases with lengths between 8 and 24 residues were chosen from studies of loop modeling and classification [23], [27], [33]–[35] that highlighted their complex but well-defined geometries (see Table 1 for full descriptions).

First, examining the subset of twenty loops used in prior PLOP and Rosetta studies permitted direct comparison of SWA to these state-of-the-art methods (see also Table S1 and Fig. S2). Here, the median Cα RMSD accuracy (lowest energy structure) was 0.64 Å, consistent with sub-Angstrom accuracy and lower than values for prior methods: 2.3 Å (PLOP [21]), 1.2 Å (PLOP with surrounding side-chain optimization [21]), 2.1 Å (Rosetta fragment assembly [22]), 1.0 Å (Rosetta KIC [22]), and 0.84 Å (Rosetta KIC repeated herein with computational power comparable to SWA calculations; Table 1 and Table S2). The SWA method thus outperforms prior loop modeling approaches.

**Figure 4:**
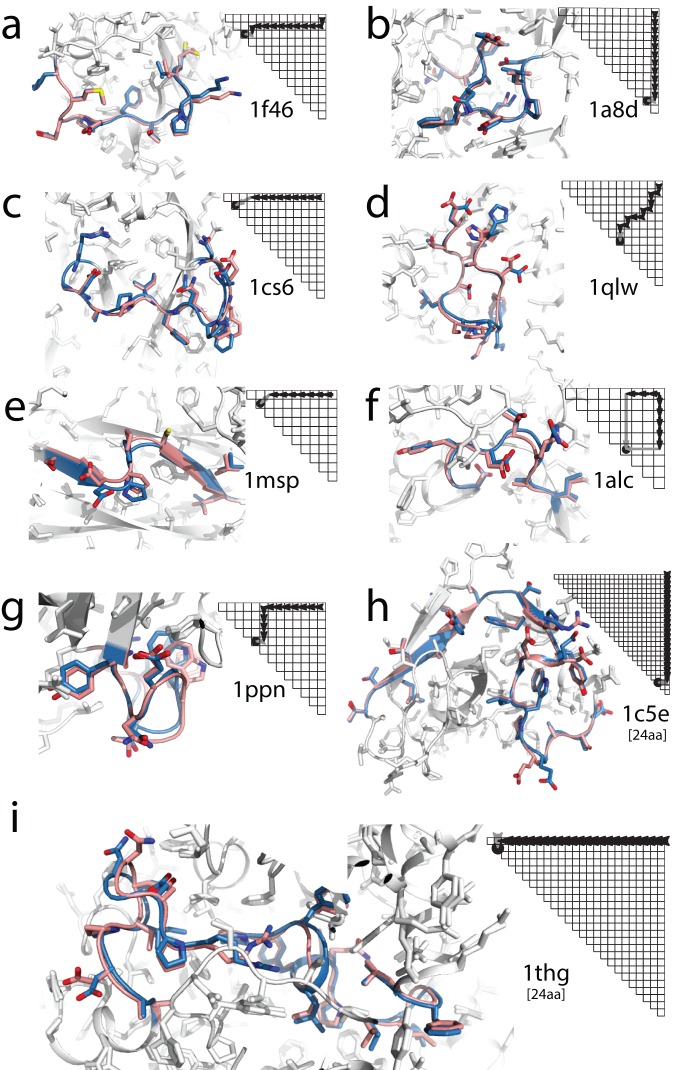
Sub-Angstrom accuracy in a benchmark of difficult protein loops by step-wise assembly. Each panel overlays the best of five lowest energy models from stepwise assembly (SWA; carbon atoms in pink) on the crystallographic loop (blue). The build-up path that gave the SWA model is shown as a dynamic-programming-style matrix (black arrows mark single-residue additions; gray arrow marks chain closure step). The Cα RMSD values achieved for each puzzle (with rank of presented model among top 5 SWA models in parentheses) were: (**a**) *1f46*, 0.46 Å (1^st^); (**b**) *1a8d*, 0.42 Å (1^st^); (**c**) *1cs6*, 0.79 Å (1^st^); (**d**) *1qlw*, 0.66 Å (3^rd^); (**e**) *1msp*, 0.73 Å (1^st^); (**f**) *1alc*, 0.58 Å (2^nd^); (**g**) *1ppn*, 0.89 Å (1^st^); (**h**) *1c5e* (24-residue), 0.41 Å (1^st^); (**i**) *1thg* (24-residue), 0.80 Å (1^st^). In (f), the top-ranked build-up path involved generation of N-terminal and C-terminal fragments separately (leftward and downward black arrow paths), followed by recombination and chain closure; see Methods.

In 19 of the 20 cases, at least one of the five lowest energy SWA models achieved sub-Angstrom accuracy. For comparison, KIC modeling achieved this level of accuracy in fewer cases, 14 of 20 (Table 1). [A smaller number of cases (11 of 20) was reported as ‘solved’ by KIC in prior work, which used less computational power (1000 CPU hours), did not sample *cis* prolines, and reported RMSD for the very lowest energy model.] The high accuracy SWA predictions that were intractable to previous PLOP and/or Rosetta approaches included the *1oyc* case described above (Fig. 2L) as well as a loop containing both a *cis* proline and *trans* proline from ZipA (*1f46*, Fig. 4A), a loop with a ‘corkscrew’ fold in tetanus toxin C (*1a8d*; Fig. 4B), a highly extended loop from an immunoglobulin domain involved in neural cell adhesion (*1cs6*, Fig. 4C), and a hairpin-like loop from a bacterial esterase (*1qlw*; Fig. 4D). The residue-by-residue paths that achieved the high accuracy SWA models were different for each case (pathway traces in Fig. 4), underscoring the necessity of following all build-up paths.

For the 15 additional test cases, SWA gave somewhat lower performance, as expected, given that these cases were selected for their complexity. SWA achieved median Cα RMSD accuracies of 1.4 Å (lowest energy structure) and 0.89 Å (best of five lowest energy structures). SWA modeling again outperformed KIC modeling overall, although not by as much as in the first 20-loop benchmark [1.9 Å (lowest energy) and 0.94 Å (best of five)]. In 8 of these 15 complex loop tests, SWA returned at least one of five lowest energy models with sub-Angstrom accuracy (Table 2; see also Fig. S3). High-resolution structures were recovered for segments that contained *cis*-Pro touch turns [35] (*1msp*; Fig. 4E), that threaded through ‘tunnels’ formed by other side chains in α-lactalbumin [21] (*1alc*, a highlighted problem case for PLOP [20], [21]; Fig. 4F), and that bound inhibitors in papain [36] (*1ppn*; Fig. 4G). KIC modeling also achieved sub-Angstrom accuracy in 8 of 15 test cases, but not in all the same cases (see below).

The most striking SWA models involved loops with long lengths (Figs. 4H and 4I). Formally, the exponential growth of possible conformations with loop size makes a 24-residue loop puzzle substantially more difficult than a 12-residue loop (with approximately 10^12^–fold more accessible conformations). However, the stepwise ansatz underlying the SWA method constrains sampling to a subspace that requires only 4-fold more steps to search. For three of the six cases with lengths greater than or equal to 18 residues, the SWA method achieved sub-Angstrom accuracy. Modeling with such accuracy included two 24-residue cases. One involved a mixture of irregular, helix, and strand segments in a bacteriophage head protein (*1c5e*; Fig. 4H), and another involved a long loop threading through the center of a lipase domain (*1thg*; Fig. 4I). For these loops, extensive KIC modeling runs (Table S2) failed to achieve any models at any energy with RMSD accuracy better than 2.0 Å (Fig. S2). These results illustrate the effectiveness of the stepwise ansatz in reducing the vast conformational space of a protein segment into a physically realistic subspace that can be systematically searched with available computational resources.

### Problem cases and the Rosetta energy function

Overall, 27 of the 35 loop puzzles were solved with atomic accuracy by the SWA method, taking into account the five lowest energy models. Most of the residual problems appeared due to inaccuracies in the assumed energy function, analogous to observations made for SWA modeling of RNA loops [13]. For example, even amongst the 27 success cases, the best of five lowest energy conformations – but not the very lowest energy conformation – achieved sub-Angstrom accuracy, suggesting imperfect energy function discrimination amongst these low energy states.

Further evidence for energy function problems came from SWA problem cases. For six of the eight cases in which sub-Angstrom accuracy was not achieved, the SWA approach uncovered non-native models with energies within 3 *k*_B_ *T* of optimized crystallographic loops (Table S2; four cases were within 1 *k*_B_ *T*). Interestingly, in two of these cases (*1arp* and *1huw*), KIC modeling outperformed SWA modeling in terms of RMSD but gave significantly worse Rosetta energies (>10 k_B_T; Table S2 and Figure S3). This comparison suggests that SWA's strong optimization of the Rosetta all-atom energy function – apparently quite inaccurate in these two cases – prevented it from sampling the full diversity of conformations discovered by the KIC method in its low-resolution stage. These two loops – as well as two of the eight problem cases in which SWA gave significantly worse energies than the experimental loop – were solvent exposed and making few non-polar interactions. Future improvements in the Rosetta energy function, particularly in its highly oversimplified solvent model [37], [38], may better guide SWA modeling in early stages to partial loop conformations that give better all-atom energies and/or accuracies.

### Blind tests

**Figure 5:**
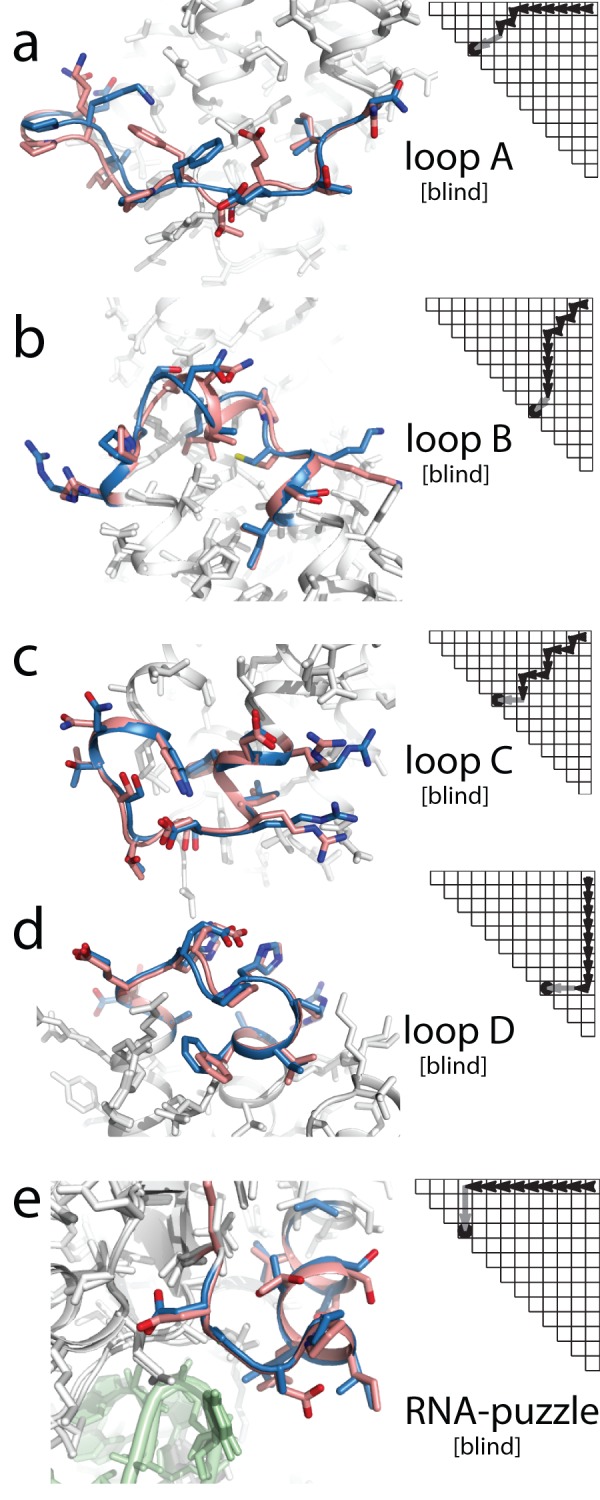
Sub-angstrom accuracy in blind structure prediction of protein loops. Each panel overlays the best of five lowest energy models from stepwise assembly (SWA; carbon atoms in pink) on the crystallographic loop (blue). The build-up path that gave the SWA model is shown as a dynamic-programming-style matrix (black arrows mark single-residue additions; gray arrow marks chain closure step). (**a**–**d)** Recovery of loops of an unreleased structure of 275-residue protein with all loops and all side-chains removed, with Cα RMSDs of 0.91 Å, 0.91 Å, 0.54 Å, 0.52 Å. (**e**) *3v7e* RNA-puzzle (RMSD 0.54 Å), with RNA component shown in green.

Blind trials with sequences of previously unknown structure provide important tests of structure prediction methods. For this study, SWA was tested on five such cases, from two sets of problems. Four problems involved the loops from a 275-residue crystallographic model of an all-α protein complexed to a long helix, recently solved by Weis and collaborators (Stanford University) and not released outside their research group. The closest previously solved structure exhibited low sequence identity to the target (26% over a 210-residue alignable region); and analogs of the loop regions either did not exist (loop A) or were different in sequence at all 12 positions (loop B) or at 10 positions (loops C and D) in the homologous structure. The Weis group provided a starting structure with all of these loops and all side-chains removed, and *ab initio* SWA models were generated for the four loops. Upon un-blinding, SWA models gave sub-Angstrom recovery in all four cases (Table 2; Figs. 5A–D), including a 0.90 Å model of loop A, which formed an irregular lasso around the protein's binding partner (Fig. 5A). In three of these four cases, KIC modeling also achieved sub-Angstrom accuracy (Table 2).

As a fifth blind test, SWA models were generated for a loop of protein YbxF [39] that interacted with the SAM-I riboswitch RNA, an RNA/protein target that constituted the fourth ‘RNA-puzzles’ trial [14]. This problem was more challenging than those above, as the starting structure was not a crystallographic model but instead a comparative model [40% sequence identity to template *2fc3*] based on threading with HHPRED and Rosetta [5], [40]. SWA modeling of the loop, including nearby RNA atoms as potential interactors, gave a conformation 2.0 Å Cα RMSD away from the loop in the starting comparative model. Nevertheless, this loop agreed with the conformation in the subsequently released structure (*3v7e*; Fig. 5E) at 0.53 Å Cα RMSD. These results demonstrate the utility of the SWA protocol in a complex structure prediction context in which the scaffold is not a crystallographic model but a comparative model. An exact comparison with KIC was not possible here due to the lack of a coarse-grained RNA/protein interaction potential in Rosetta; however, KIC modeling of the protein loop without the RNA also returned a sub-Angstrom accuracy loop (0.80 Å Cα RMSD), albeit as the second lowest energy model.

## Discussion

### A novel and systematic strategy for protein structure modeling

This article has presented a strategy for protein structure prediction that achieves atomic accuracy on the majority of loop modeling targets through a systematic all-atom enumeration. Several of these targets were difficult or intractable with prior approaches, despite mainly involving loops excised from crystallographic models, the simplest such puzzles. The main innovation herein is a stepwise ansatz imported from RNA structure modeling. This working hypothesis posits that realistic loop structures are reachable via the residue-by-residue building of partial conformations that are themselves well-stabilized by precise hydrogen bonds and non-polar packing interactions. This ansatz underlies a Rosetta stepwise assembly (SWA) protocol and is supported by tests of the SWA algorithm on forty loop puzzles, including twenty shared with prior loop modeling benchmarks, fifteen more difficult loop cases, and five blind tests. In the majority of cases (32 of 40), including loop puzzles of unprecedented length, all the blind tests, and a comparative model (rather than a crystal structure), the SWA method achieved sub-Angstrom accuracy.

The stepwise assembly protocol is novel in protein modeling studies: while prior efforts have proposed the build-up of short peptides or lattice models [21], [41]–[44], the SWA method herein provides a complete enumerative protocol without coarse-graining, stochastic search, or other approximations. This study has demonstrated that such calculations are achievable for a 12-residue protein loop in approximately 5,000 CPU-hours, readily accessible with modern parallel computing clusters. This expense is greater than Monte Carlo or refinement-based approaches, which, in favorable cases, can recover loop conformations in hundreds of CPU-hours or less [21], [22], including some of the challenges considered herein. Nevertheless, many complex loops remain unsolvable by these prior approaches, even with the expenditure of massive computational power (Table 2 and Table S1). Thus, for the general case, the computational expense of SWA may be worthwhile. Future optimizations in continuous minimization of protein configurations and in criteria to prune build-up paths, are expected to accelerate the method, as well as incorporation of sparse experimental constraints (see below).

### An analogy to another enumerative solution: lock-picking

**Figure 6:**
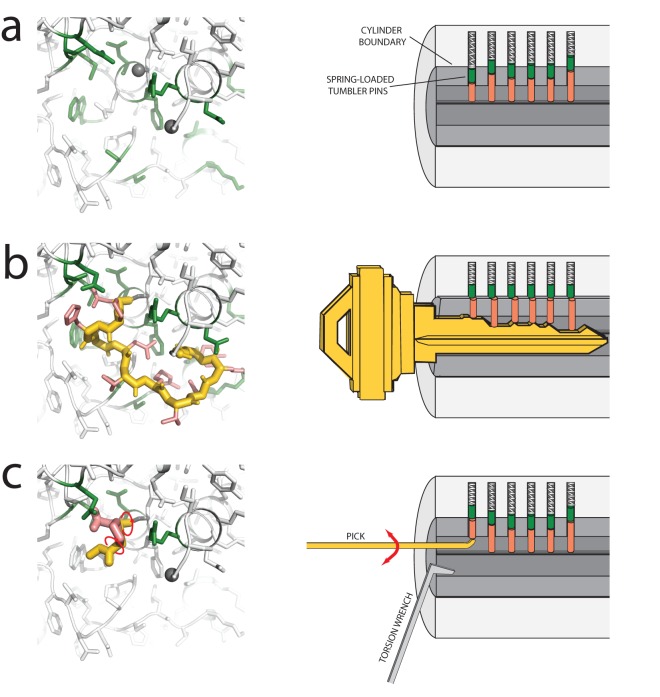
Lock-picking analogy for high-resolution protein loop structure prediction. (**a**) Starting conformation for loop structure prediction (left panel) includes the crystallographic backbone outside the excised loop (white; gray spheres mark loop boundaries) and side-chains in potentially incorrect configurations (white and green). The situation is analogous to a pin tumbler lock (right panel) with spring-loaded pin doublets (green & pink cylinders) in relaxed configurations that disallow turning of the lock cylinder (gray, in cut-away view). (**b**) Atomic-accuracy solution for protein loop (left panel) involves precise fit of loop backbone (gold) and side-chains (pink) to surrounding protein. Several surrounding side-chains (green) have switched conformations. The solution is analogous to a key (right panel) that precisely slides up the pin doublets so that the boundaries between top and bottom pins coincide with the boundary of lock cylinder, which can then be turned. (**c**) ‘Stepwise ansatz’ involves residue-by-residue build-up of the protein loop (gold and pink), with fine, all-atom enumerative search of loop conformations (red arrows mark some of the backbone torsions searched) and surrounding side-chains (green) at each step. The strategy is analogous to picking a tumbler lock with a probing pick (gold, right panel) that finely sets the tumbler pins into the turnable configuration through pin-by-pin fine search. [A wrench (light gray) applying torsion to the cylinder helps trap each pin at the boundary].

An analogy to lock-picking helps clarify the strengths and limitations of the stepwise assembly method described herein. The main innovation of the protein SWA method is the enumerative sampling of individual residue conformations, which helps guarantee precise fit of the residues' atoms into the surrounding environment. This scenario is a kind of ‘lock-and-key’ problem but differs from the previous RNA SWA method [13] in that the surrounding side chains are not fixed. In that sense, protein loop modeling is less of a ‘lock-and-key’ problem than a problem closer to the picking of a house-hold tumbler lock, which has several sets of pin doublets that move in response to a key or pick (Figs. 6A and 6B). In this case, finding the key for the lock is analogous to modeling the protein loop given the backbone of the surrounding scaffold; and the lock’s moving pins are analogous to the moving (and previously unknown) side-chains in the protein modeling problem.

To unlock a tumbler lock, one ‘enumeratively samples’ each pin conformation through manipulation with a probing pick, until the boundaries within the pin doublet are aligned with the lock's cylindrical surface (Figure 6C). The pin doublets are trapped at the surface through the continuous application of torque. This picking is analogous to the enumerative sampling of individual protein loop residues and optimization of surrounding side-chains during stepwise assembly. After the entire set of lock pins has been picked one-by-one, the pins line up precisely with the lock cylinder boundary, analogous to the close fit of the native protein loop with its surroundings, and the lock cylinder can be turned.

This analogy clarifies the importance of precise fits between the loop and its surroundings for successful modeling; if such a fit is not possible, the current SWA implementation will give inaccurate solutions. In particular, SWA will have difficulty in problems where the given protein backbone (outside the loop) deviates from its actual conformation. Such scenarios are encountered in comparative modeling and protein design applications. Cases like the community-wide blind trial YbxF above (Fig. 5E) demonstrate that SWA can succeed in realistic comparative modeling scenarios with small backbone shifts. Nevertheless, in more complex cases, extensions of SWA that permit minimization of the surrounding backbone will need to be explored.

### Scaling with problem size

Given that SWA carries out a systematic residue-by-residue enumeration, it is perhaps surprising that it remains computationally efficient for long loops. For a protein segment with length of *N* residues, the formal size of the conformational space scales exponentially with *N*; the actual experimental folding times of proteins do not scale so poorly, implying the general existence of folding intermediates or pathways rather than a random walk search [1]. In SWA, the number of steps required to build a protein loop does scale efficiently (in polynomial time) with *N*. The resulting efficiency permitted the atomic resolution recovery herein of loops with lengths up to 24 residues, whereas prior work tackled loops no longer than 12 residues (see, e.g., [21]–[23]). The scaling efficiency of SWA suggests that *ab initio* buildup of full proteins, and not just loops, should be possible; preliminary work has demonstrated the feasibility of modeling mini-proteins with lengths of 30 residues [33].

### Implications for protein folding

The general success of the SWA modeling method suggests that the lowest-energy *in silico* pathways of the calculation (see, e.g., Figs. 3 and 4) may represent actual *in vitro* folding pathways of a protein loop. In this case, the *in silico* ‘instantiation’ of each protein residue during SWA might be thought of as the *in vitro* formation of a fixed structure by that residue, which was originally in a random-coil or transiently structured ensemble of conformations. Development of a kinetic model from the SWA-calculated energy landscape (analogous to efforts in RNA secondary structure [45]) as well as increases in spatial and time resolution of experimental single-molecule approaches to protein folding may be able to test these predicted folding pathways.

### Implications for practical structural biology

The SWA algorithm for protein loop modeling presented herein may have unique and practical uses in several areas of structural biology. In *ab initio* structure modeling problems, as discussed above, SWA offers the potential for consistent sub-Angstrom accuracy as well as a powerful (if computationally demanding) tool for uncovering deficiencies in the Rosetta energy function, as in the solvent-exposed loops described above (see also [33]). In addition, there are numerous practical applications of *ab initio* modeling that do not rely on a ‘perfect’ energy function and instead make use of limited experimental data to break degeneracies. These problems include the high-resolution fitting of coordinates into low-resolution electron density maps [8], the solving of NMR structures from sparse chemical shift data [9], [46], and the determination of ‘invisible state’ structures from both NMR and crystallographic approaches [47], [48]. As with analogous RNA problems in which SWA is proving to be uniquely powerful [15], [16], [18], [49], the protein SWA method herein is expected to be substantially accelerated in these use cases due to the inclusion of experimental constraints and to give models with a particularly high level of confidence due to the algorithm’s guarantee of enumeration. Finally, efforts to design protein interfaces and enzyme active sites often encounter sampling bottlenecks due to the difficulty of simultaneously optimizing side-chain identity, side-chain conformation, and new backbones [3]. If expanded to include residue-by-residue side-chain identity optimization, the stepwise ansatz may offer an efficient working hypothesis for designing such functional molecules by enumeration.

## Methods

Stepwise assembly (SWA) was implemented in C++ in the Rosetta codebase and is available in Rosetta release 3.6, free to academic users at http://www.rosettacommons.org. Descriptions of the sampling method, the directed acyclic graphs for entire calculations, and explicit command-line examples, are given in the following sections.

### Stepwise Assembly (SWA)

A diagram of the entire stepwise assembly (SWA) calculation is given as a directed acyclic graph (DAG) laid out in the style of a dynamic programming matrix in Figure 1D. Given a crystallographic loop to be built *de novo* from residue *k* to residue *l*, each stage of stepwise assembly involved creating models of the loop with an N-terminal fragment built forward from *k*–1 to residue *i* and a C-terminal fragment built backward from residue *l*+1 to *j*. Each stage could thus be indexed with the two residue positions (*i*,*j*). The SWA calculation proceeded recursively from stages with short fragments built into the structure towards models with longer fragments, i.e., *i* increasing from *k*–1 or *j* decreasing from *l*+1. This building corresponds to movement from the top-right to the bottom or left, respectively, in Fig. 1D. The SWA calculation involved five basic kinds of steps (see next section for example Rosetta command lines):

#### (1) Pre-packing of the starting model

The first step was a ‘pre-packing’ of the side-chains of the starting model with no loop atoms, corresponding to the top-right corner (*k*–1, *l*+1) of the DAG in Fig. 1D. This step was necessary as our starting models contained no side chains as well as no loop atoms. This prepacking stage thus placed initial side-chains (constructed with Rosetta ideal bond lengths and angles) using the Rosetta *packer*. This pack optimized the rotamers using simulated annealing, after precomputing pairwise energies between all potential side-chain rotamers [28]. After packing, the side-chain torsions were subjected to the non-monotone Armijo variant of Broyden-Fletcher-Goldfarb-Shanno (BFGS) minimization using the Rosetta *minimizer* [38], [50]. All non-loop backbone degrees of freedom were held fixed here and in all later stages of the SWA calculation.

#### (2) Addition of single residue to N-terminal fragment of the loop

The core computation in SWA is the addition of a new residue to a model and enumeration of its backbone conformations. For additions to the *N*-terminal fragment, this step took models from stage (*i*–1, *j*) to (*i*, *j*) (downward arrows in Fig. 1D). The newly added residue included a methylamide group at the C-terminus, simulating the peptide connection to the next residue (including the next backbone amide; Fig. 1A). The enumeratively sampled degrees of freedom were backbone torsions for both the added residue (φ*_i_* and ψ*_i_*) and the previous, adjacent residue (φ *_i_*_–1_, ψ*_i_*_–1_), permitting the discovery of configurations in which the dipeptide segment is stabilized by interactions by the new residue without requiring interactions at the previous residue. (For the initial loop residue, *I* = *k* the previous residue torsions were fixed and not sampled). For each φ or ψ, the sampling was a grid search from –180° to 180° in 20° increments. To keep only sterically realistic backbones, configurations in which a residue's (φ,ψ) gave Rosetta *ramachandran* score greater than 0.8 Rosetta units were discarded. The ω torsion was assumed to be 180° (*trans* configuration), except for residues that preceded prolines, which were also sampled at 0° (*cis*). The number of backbone combinations varied from tens to several thousand (for segments involving glycine residues and/or residues that preceded proline).

For each combination of backbone torsion angles, the side-chains of the loop and its surroundings were optimized. Analogous to calculations in protein-protein docking [24], [51], the side-chain optimization was focused on the two residues whose backbones were sampled (*i* and *i–*1) and their potential neighbors (the *neighbor_list*, determined based on Cβ locations by Rosetta scoring). The side-chain sampling was carried out with the Rosetta *rotamer_trials* algorithm. (Runs using the more computationally intensive *packer* algorithm gave indistinguishable results; the problem involves few side chains and the optimum is found easily.) The searched side-chain rotamers included those listed in the backbone-dependent Rosetta rotamer library as well as additional rotamers with χ_1_ and χ_2_ shifted by ±1 standard deviation from the standard rotamer values. The discreteness of the backbone grid search and rotamer library can penalize favorable side-chain interactions due to minor clashes or slightly imperfect hydrogen bonds. Therefore, the energy function for side-chain optimization was modified from the current standard Rosetta all-atom energy function (*score12*) to include a lower weight on *fa_rep* (Lennard-Jones repulsion; 0.10 instead of 0.44), a higher weight on *hbond_sc* (side-chain/side-chain hydrogen bond strength; 3.1 instead of 1.1), and no attenuation of hydrogen bond strength at solvent-exposed residues [38], [52].

After enumerative backbone sampling and side-chain optimization, models were clustered as follows. In order of energy, starting with the lowest energy model, the RMSD of each model to all lower energy clusters was computed; this RMSD value was calculated over N, C, Cα, and O atoms at the rebuilt residues *i*–1 and *i* (all other residues shared the same backbone configuration). If the RMSD value to any lower energy clusters was less than a fine cutoff (0.10 Å), the model was considered too close to an existing representative and discarded; otherwise the model seeded a new cluster. The lowest energy 400 models after clustering were carried forward to minimization.

Minimization involved backbone torsions (φ, ψ, and ω) at the sampled residue and torsions χ for all neighboring side-chains (Rosetta ideal bond lengths and angles were assumed as fixed throughout the SWA procedure). This torsional optimization was performed with the non-monotone Armijo variant of BFGS minimization in the Rosetta *minimizer* [38], [50]; the energy function was the current standard Rosetta all-atom energy function *score12*. The models were clustered as described above, and saved to disk.

#### (3) Addition of single residue to C-terminal fragment of the loop

The ‘prepending’ of a new residue *j* to the C-terminal loop fragment (leftward arrows in Fig. 1B) was analogous to the step appending to the N-terminal fragment above. An acetyl group at the N-terminus of *j* simulated a peptide connection to the previous residue (Fig. 1B). Residues *j* and *j*+1 were subjected to enumerative backbone search, side-chain packing, model clustering, torsional minimization, and final clustering as above.

#### (4) Chain closure

For the final stages of SWA assembly, N-terminal and C-terminal fragments were bridged to form continuous loops with ideal backbone bond lengths and angles (Figure 1C), and the entire resulting loops were subjected to continuous minimization. Chain closure attempts were carried out for all models in which the number of gap residues between the N-terminal and C-terminal fragments was 1, 2, or 3 [that is, for models from stage (*i*, *j*) where 1< (*j*–*I*–1) <3].

As preparation for chain closure, the N-terminal gap residue *i*+1 was appended to the N-terminal fragment, and other gap residues *i*+2 to *j*–1 were prepended to the C-terminal fragment. The φ and ψ torsion angles of the first gap residue *i*+1 were sampled by grid search as above; and, to attempt chain closure, backbone torsions for ‘bridge’ residues *i*+2 up to *j*–1 were subjected to 1000 cycles of cyclic coordinate descent [CCD; *fast_ccd_loop_closure* in Rosetta [28], [53]]. CCD was applied to all φ and ψ torsion angles at bridge residues; on the ψ torsion immediately preceding the first bridge residue (here on the other side of the chainbreak); and on the φ torsion immediately after the last bridge residue. Any models with chain closure RMSD (see below) less than 1.5 Å were then passed forward to side-chain optimization, clustering, and then continuous torsional minimization as above, except that all loop side-chains (not just newly built residues) and all loop backbone torsions were optimized in these steps, along with neighboring side-chains from the surrounding protein scaffold. The last full-loop torsional minimization offered an opportunity to improve closure geometry. Rosetta chain closure involves three virtual N, C, and Cα atoms prepended or appended to the residue immediately after or before the chainbreak, respectively; these virtual atoms should perfectly overlap with the actual N, C, and Cα atoms in the case of exact chain closure (chain closure RMSD of zero). Here, the Rosetta *linear_chainbreak* term, equal to sums of these atom-atom deviations (in Å), was applied with a strong weight of 150.0. The inclusion of this pseudo-energy term ensured that chains closed by CCD retained or improved near-perfect geometries during full-loop minimization, with typically less than 0.01 Å deviations from perfect closure in final, lowest energy models.

In addition to the chain closure protocol described above, which enumeratively samples the first ‘gap’ residue and CCD-closes the rest, all models were subjected to an analogous protocol carrying out backbone grid search at the last gap residue *j*–1, and closing the chain by CCD optimization of φ and ψ at the preceding residues.

Finally, closed-loop models were also prepared by combining all models for just N-terminal fragments (*i*, *l*+1) and all models for just C-terminal fragments (*k*–1, *j*); combining these fragments gave models equivalent to those from stage (*i*, *j*) that were subjected to the same chain closure, full-loop side-chain optimization, and full-loop torsional optimization as above. Loop closure steps based on analytical kinematic closure were also investigated but gave fewer successful closures than the CCD-plus-minimization approach above.

#### (5) Clustering of models

For a given build-up stage (*i*, *j*), up to 400 models were generated from each of 400 models at previous build-up stages [(*i*–1, *j*) and (*i*, *j*+1)], leading to hundreds of thousands of models. Even larger numbers were generated at the final stage of full-length loop modeling due to the many routes to chain closure. However, these models typically spanned a very large range of energies, and SWA seeks to carry forward only the lowest-energy configurations at each rebuild stage. Thus all models for a given stage were collated, filtered to retain the 4000 lowest energy models, and then reclustered. The clustering followed the procedure described above, except that RMSDs were calculated over the entire rebuilt loop fragments and a clustering RMSD threshold of 0.25 Å was applied. The 400 lowest energy configurations were carried forward. In the final stage (full-length loop models), models were re-clustered with RMSD threshold 1.0 Å, and the five lowest energy models were taken as the SWA predictions.

In the SWA runs for this study's 35-loop benchmark, some settings in the loop modeling were chosen so as to match prior benchmarks. First, for proteins containing disulfide bonds, these residue-residue pairings were assumed to be known [as in prior work [22]]; those cysteine residues were modeled without protonation of the sulfur and using standard Rosetta terms that favored disulfide bonding with typical bond lengths, angles, and torsions [54]. Second, starting structures were taken directly from the previously studied benchmark set [for the 20-protein PLOP/Rosetta test set [21], [22]] or prepared by addition of hydrogens with Reduce [55], removal of all side-chain atoms, and excision of loop regions. Third, the amide H atom at the C-terminal loop endpoint (*l+*1) is coupled to the loop conformation through the torsion φ*_l+1_.* This degree of freedom was not sampled, as ‘native’ backbone hydrogen atoms were present in the benchmark starting structures. However, for the five blind tests, the hydrogens were not available *a priori*; they were initially placed in the starting excised structure with Reduce [55], and since the amide H atom at position *l+*1 was not guaranteed to be in its ‘native’ position, φ_l+1_ was sampled during build-up of residues *l*–1 and *l*.

It was found empirically in early tests on small loops (6–9 residues) that some cases could be solved without carrying out the full SWA dynamic programming matrix, but instead by building from the N-terminal side (without C-terminal growth), by building in separate runs from the C-terminal side (without N-terminal growth), and then combining these separate solutions with chain closure to attain final models. This simplified calculation (outlined in Figure 1E) grows as O (*N*) with the number of residues *N*, rather than as O (*N* ^2^), and is analogous to the recursion used previously for RNA loop modeling [56]. For all cases in this study, this O (*N*) calculation was carried out first. If the energy gap between the lowest energy model and the second lowest energy model was less than 1 *k*_B_ *T*, the calculation was assumed to not have clearly converged on a confident model, and the loop building was repeated with the full O (*N* ^2^) calculation, except the very long *1rhd* and *7cat* test cases. Overall, 18 of 40 cases were modeled with the O (*N*) calculation (see Table 2).

### SWA example command lines

The entire SWA calculation can be set up with the following Python script, available in the Rosetta subdirectory tools/SWA_protein_python/:

- generate_swa_protein_dag.py -loop_start_pdb noloop_1oyc_min.pdb -native 1oyc_min.pdb -fasta 1oyc.fasta -cluster_radius 0.25 -final_number 400 -denovo 1 -loop_res 203-213 214 –weights score12.wts -disable_sampling_of_loop_takeoff -loop_force_Nsquared

here, noloop_1oyc_min.pdb is the starting structure. The -native flag is optional and specifies a reference PDB can be supplied to compute RMSD values for the loop during the calculation. 1oyc.fasta gives the sequence of the entire protein in FASTA format:

- >1oyc.pdb SFVKDFKPQALGDTNLFKPIKIGNNELLHRAVIPPLTRMRALHPGNIPNRDWAVEYYTQRAQRPGTMIITEGAFISPQAGGYDNAPGVWSEEQMVEWTKIFNAIHEKKSFVWVQLWVLGWAAFPDNLARDGLRYDSASDNVFMDAEQEAKAKKANNPQHSLTKDEIKQYIKEYVQAAKNSIAAGADGVEIHSANGYLLNQFLDPHSNTRTDEYGGSIENRARFTLEVVDALVEAIGHEKVGLRLSPYGVFNSMSGGAETGIVAQYAYVAGELEKRAKAGKRLAFVHLVEPRVTNPFLTEGEGEYEGGSNDFVYSIWKGPVIRAGNFALHPEVVREEVKDKRTLIGYGRFFISNPDLVDRLEKGLPLNKYDRDTFYQMSAHGYIDYPTYEEALKLGWDKK.

The numbering of the loop residues takes the convention that the first amino acid in the sequence starts at 1. Note that these numbers can be offset from the starting PDB numbering (Table 1).

Simplified calculations that take O(*N*) steps with the number of loop residues *N* (see above) are setup with the same swa_protein_dagman.py script above, but without the last flag –loop_force_Nsquared. For blind tests, the ψ torsion for the starting loop residue and φ torsion for the ending loop residue were sampled (see above); this was accomplished by omitting the flag -disable_sampling_of_loop_takeoff.

Upon running swa_protein_dagman.py, the entire calculation workflow is laid out in one text file describing the directed acyclic graph, protein_build.dag. This master job file is in the standard CONDOR DAGMAN [57] format, and specifies the jobs and their dependencies, locations of CONDOR submission files for each job, and pre- and post- processing script commands. The entire workflow can then be carried out with CONDOR's condor_dagman. Alternatively, the workflow has also been carried out on clusters with Load Sharing Facility (bsub), Portable Batch System (qsub), and MPI job management systems. Python scripts optimized for each of these job management systems are being made available in tools/SWA_protein_python/, initiated with SWA_dagman_continuous.py.

The CONDOR submission files, each containing the appropriate Rosetta command line, are created in a CONDOR/subdirectory. Additional scripts specified for pre-processing CONDOR submission files (to reflect the number of models at each stage) and for post-processing all models available for a given stage by collation into single files and removal of unused files, are available in tools/SWA_protein_python/.

Each of the build steps described in protein_build.dag corresponds to a single command line using the Rosetta executable swa_protein_main.

1. An example command-line for pre-packing the 1OYC loop modeling case is:
  - swa_protein_main.<exe> -database <path to database> -rebuild -out:file:silent_struct_type binary -fasta 1oyc.fasta -n_sample 18 -nstruct 400 -cluster:radius 0.100 -extrachi_cutoff 0 -ex1 -ex2 -score:weights score12.wts -pack_weights pack_no_hb_env_dep.wts -in:detect_disulf false -add_peptide_plane -native 1oyc_min.pdb -superimpose_res 1-202 215-399 -fixed_res 1-202 215-399 -calc_rms_res 203-214 -jump_res 1 399 -disable_sampling_of_loop_takeoff -mute all -s1 noloop_1oyc_min.pdb -input_res1 1-202 215-399 -use_packer_instead_of_rotamer_trials -out:file:silent REGION_215_202/START_FROM_START_PDB/region_215_202_sample.out
2. An example command line that builds residue 206 onto the end of a N-terminal fragment already containing 203–205 is:
  - swa_protein_main.<exe> -database <database> -rebuild -out:file:silent_struct_type binary -fasta 1oyc.fasta -n_sample 18 -nstruct 400 -cluster:radius 0.100 -extrachi_cutoff 0 -ex1 -ex2 -score:weights score12.wts -pack_weights pack_no_hb_env_dep.wts -in:detect_disulf false -add_peptide_plane -native 1oyc_min.pdb -superimpose_res 1-202 215-399 -fixed_res 1-202 215-399 -calc_rms_res 203-214 -jump_res 1 399 -disable_sampling_of_loop_takeoff -mute all -silent1 region_215_205_sample.cluster.out -tags1 S_0 -input_res1 1-205 215-399 -sample_res 205 206 -out:file:silent REGION_215_206/START_FROM_REGION_215_205_DENOVO_S_0/region_215_206_sample.out

Here, the build is onto the lowest energy model (S_0) available from a previous stage that had rebuilt residues 203–205 from the N-terminal end.

1. An example command line that builds residue 209 onto the N-terminal end of a C-terminal fragment already containing residues 210–214:
  - swa_protein_main.<exe> -database <path to database> -rebuild -out:file:silent_struct_type binary -fasta 1oyc.fasta -n_sample 18 -nstruct 400 -cluster:radius 0.100 -extrachi_cutoff 0 -ex1 -ex2 -score:weights score12.wts -pack_weights pack_no_hb_env_dep.wts -in:detect_disulf false -add_peptide_plane -native 1oyc_min.pdb -superimpose_res 1-202 215-399 -fixed_res 1-202 215-399 -calc_rms_res 203-214 -jump_res 1 399 -disable_sampling_of_loop_takeoff -mute all -silent1 region_210_202_sample.cluster.out -tags1 S_2 -input_res1 1-202 210-399 -sample_res 209 210 -out:file:silent REGION_209_202/START_FROM_REGION_210_202_DENOVO_S_2/region_209_202_sample.out

Here, the build is onto the third lowest energy model (S_2) available from a previous stage that had rebuilt residues 210-214 from the C-terminal end.

1. An example command line that takes the lowest energy model from the ensemble that has built up both the N-terminal fragment 203–206 and C-terminal fragment 209–215, samples backbone degrees of freedom at residue 207, and closes the chain by cyclic coordinate descent (CCD) across residues 207 and 208:
  - swa_protein_main.<exe> -database <path to database> -rebuild -out:file:silent_struct_type binary -fasta 1oyc.fasta -n_sample 18 -nstruct 400 -cluster:radius 0.100 -extrachi_cutoff 0 -ex1 -ex2 -score:weights score12.wts -pack_weights pack_no_hb_env_dep.wts -in:detect_disulf false -add_peptide_plane -native 1oyc_min.pdb -superimpose_res 1-202 215-399 -fixed_res 1-202 215-399 -calc_rms_res 203-214 -jump_res 1 399 -disable_sampling_of_loop_takeoff -mute all -silent1 region_209_206_sample.cluster.out -tags1 S_0 -input_res1 1-206 209-399 -sample_res 208 -bridge_res 207 -cutpoint_closed 207 -ccd_close -global_optimize -out:file:silent REGION_207_206/START_FROM_REGION_209_206_CLOSE_LOOP_CCD_S_0/region_207_206_sample.out

An example command line that combines the lowest energy model for N-terminal fragment 203–206 with every model for C-terminal fragment 209–215 (built separately from each the N-terminal fragment) and carries out CCD (cyclic coordinate descent) chain closure across 207 and 208:

1.
  - swa_protein_main.<exe> –database <path to database> -rebuild -out:file:silent_struct_type binary -fasta 1oyc.fasta -n_sample 18 -nstruct 400 -cluster:radius 0.100 -extrachi_cutoff 0 -ex1 -ex2 -score:weights score12.wts -pack_weights pack_no_hb_env_dep.wts -in:detect_disulf false -add_peptide_plane -native 1oyc_min.pdb -superimpose_res 1-202 215-399 -fixed_res 1-202 215-399 -calc_rms_res 203-214 -jump_res 1 399 -disable_sampling_of_loop_takeoff -mute all -silent1 region_209_202_sample.cluster.out -tags1 S_0 -input_res1 1-202 209-399 -silent2 region_215_206_sample.cluster.out -input_res2 1-206 215-399 -bridge_res 207 208 -cutpoint_closed 206 -ccd_close -global_optimize -out:file:silent REGION_207_206/START_FROM_REGION_209_202_REGION_215_206_CLOSE_LOOP_CCD_S_0/region_207_206_sample.out
2. An example command line for clustering the lowest energy 4000 models available for the N-terminal fragment 203–205:
  - swa_protein_main.<exe> -cluster_test -silent_read_through_errors -in:file:silent REGION_215_205/start_from_region_215_204_denovo_sample.low4000.out -in:file:silent_struct_type binary -database <path to database> -cluster:radius 0.25 -calc_rms_res 203-214 -out:file:silent region_215_205_sample.cluster.out -nstruct 400 -score_diff_cut 10.000 -working_res 1-205 215-399

### Optimization of crystallographic loops

To help assess efficiency of conformational sampling, the all-atom Rosetta energies of crystallographic loops were obtained through two strategies. Generally, crystallographic loops contain minor steric clashes that are penalized by the Rosetta energy function, and these conformations need to be subjected to local optimization to permit comparison to *de novo* models, with the same bond lengths and angles as used in the modeling.

The first ‘idealize-and-optimize’ strategy mimicked that from ref. [22]. To ensure that the loop contained the same idealized bond lengths and angles as SWA or KIC modeling, the entire crystallographic structure was subjected to Rosetta-based idealization (with the *idealize* application), and the resulting idealized loop conformation was grafted into the same side-chain pre-packed structure as used in the SWA runs above. The loop and all its neighbors were subjected to combinatorial optimization by the *packer* and then all loop torsions and all side-chain torsions in the loop and surrounding residues were subjected to continuous minimization as above. As above, keeping the backbone outside the loop residue rigorously fixed requires a formal chainbreak within the loop, which remains closed during minimization due to the linear_chainbreak term. For each potential chainbreak location (immediately N-terminal to the loop, and after each loop residue), 20 runs were carried out. The command line used was the following:

swa_protein_main.<exe> –database <path to database> -rebuild -out:file:silent_struct_type binary -fasta 1oyc.fasta -n_sample 18 -nstruct 400 -extrachi_cutoff 0 -ex1 -ex2 -score:weights score12.wts -pack_weights pack_no_hb_env_dep.wts -in:detect_disulf false -add_peptide_plane -native 1oyc_min.pdb -superimpose_res 1-202 215-399 -fixed_res 1-202 215-399 -calc_rms_res 203-214 -jump_res 1 399 -disable_sampling_of_loop_takeoff -silent1 region_215_202_sample.cluster.out -tags S_0 -input_res1 1-202 215-399 -cutpoint_closed 214 -global_optimize -out:file:silent MINIMIZE_NATIVE/12/1oyc_minimize_native.out -cluster:radius 0.0 -s2 1oyc_min_idealize.pdb -input_res2 202-215 -slice_res2 202-215

A second ‘native SWA’ strategy was used to optimize the loop conformation around the crystallographic loop. In this strategy, the entire SWA calculation after the initial side-chain prepacking was repeated, but at each sampling step, models were only carried forward if their backbone RMSD to the crystallographic loop was less than 2.0 Å. In addition, Rosetta coordinate constraints at loop Cα atoms were implemented with the following Python script command line:

generate_CA_constraints.py 1oyc.pdb –cst_res 203-214 –coord_cst –anchor_res 1 –fade > 1oyc_coordinate2.0.cst

The script is available in tools/SWA_protein_python/. These constraints applied a penalty for each Cα atom deviating further than 2.0 Å from the crystallographic position, rising to a maximum of 10.0 kBT for deviations of 4.0 Å; the functional form was a cubic spline with zero derivative at 2.0 Å and 4.0 Å (the fade function in Rosetta). These constraints were activated in SWA runs by including flags -rmsd_screen 2.0 and -cst_file 1oyc_coordinate2.0.cst in swa_protein_main command lines above. In all tested loops, the SWA-native strategy gave models within 1.0 Å Cα RMSD to the crystallographic loop with lower energies than the idealize-and-optimize strategy (Figures S2 and S3); the SWA-native values are thus reported in Table S2.

### Kinematic Closure (KIC) Monte Carlo

To permit comparison of the SWA approach to a prior state-of-the-art method, the KIC (kinematic closure) loop modeling method in Rosetta was repeated on the 20-protein PLOP/Rosetta benchmark. The following command line was used:

  - loopmodel.<exe> -database <path to database> -loops:remodel perturb_kic -loops:refine refine_kic -loops:input_pdb region_FINAL.out.1.pdb -in:file:native 1oyc_min.pdb -loops:loop_file 1oyc.loop -loops:max_kic_build_attempts 10000 -in:file:fullatom -out:file:fullatom -out:prefix 1oyc -out:pdb -ex1 -ex2 -ex1aro -extrachi_cutoff 0 -out:nstruct 1000 -out:file:silent_struct_type binary -out:file:silent 1oyc_kic.out -fix_ca_bond_angles -kic_use_linear_chainbreak -allow_omega_move -sample_omega_at_pre_prolines.

The command line is identical to that used previously, except for some additional terms to ensure a fair comparison to the SWA modeling above. The flag -fix_ca_bond_angles retains N–Cα–C bond angles at ideal values defined by Rosetta; sampling these angles did not improve accuracy in prior work [22]. The flag -kic_use_linear_chainbreak uses the same linear_chainbreak penalty for chain closure as in the SWA runs; the original chainbreak term was found to give unacceptable deviations at chainbreaks in some SWA and KIC cases. The flag -allow_omega_move activates minimization at ω residues and -sample_omega_at_pre_prolines activates the sampling of cis proline configurations during KIC Monte Carlo moves, both matching treatment of ω torsions in the SWA approach above.

The KIC loop modeling method requires an input starting structure with a loop pre-built, and this loop defines the fixed bond lengths and angles used in the run. Rather than using the crystallographic loops [22], this study used the lowest energy model achieved in SWA modeling above. This starting point ensured that side-chains distant from the loop were in the same conformation in the KIC and SWA runs (global re-packing of those side-chains otherwise introduces noise in energy comparisons), and that exactly the same bond geometries were used in KIC and SWA runs.

## Supporting Information

Figure S1 **Energy vs. RMSD plots at intermediate stages of stepwise assembly build-up.** Build-up in panels (a) to (k) corresponds to residue-by-residue path in Figs. 1A–K of main text for the *1oyc* loop (residues 203–214). RMSD over all backbone heavy-atoms (N, C, Cα, and O) is shown on x-axis, using the corresponding loop fragments in the crystallographic loop as a reference. In each panel, symbol with black outline marks the specific model that eventually leads to the final lowest energy model in (k).
(EPS)

Figure S2 **Energy vs. RMSD summaries of modeling runs for 20-loop PLOP/Rosetta benchmark.** Rosetta all-atom energy values and loop Cα RMSDs are plotted for models from kinematic closure Monte Carlo (KIC, gray); stepwise assembly with O(*N*) simple calculation (red); stepwise assembly with full O(*N* ^2^) build-up (pink); crystallographic loops optimized by SWA re-building with constraints (blue); and crystallographic loops optimized by idealizing, re-packing, and continuous minimization (green).
(ZIP)

Figure S3 **Energy vs. RMSD summaries of modeling runs for 15 difficult and 5 blind test cases.** Rosetta all-atom energy values and loop Cα RMSDs are plotted for models from models from kinematic closure Monte Carlo (KIC, gray); stepwise assembly with O(*N*) simple calculation (red); stepwise assembly with full O (*N* ^2^) build-up (pink); crystallographic loops optimized by SWA re-building with constraints (blue); and crystallographic loops optimized by idealizing, re-packing, and continuous minimization (green). For 3v7e (blind test, RNA-binding protein ybxF), optimized crystallographic energies are not presented, since loop building was carried out on a comparative model; and energies between KIC and SWA cannot be compared as RNA was not included in the former case.
(ZIP)

Table S1 **Comparison of all loop modeling methods in 20-residue PLOP/Rosetta benchmark.**
(PDF)

Table S2 **Energy comparisons to determine convergence and conformational sampling efficiency.**
(PDF)

## References

[1] Levinthal C (1968) Are there pathways for protein folding. Journal de Chimie Physique et de Physico-Chemie Biologique 65: 44–45.

[2] Fleishman SJ, Baker D (2012) Role of the biomolecular energy gap in protein design, structure, and evolution. Cell 149: 262–273. 22500796 10.1016/j.cell.2012.03.016

[3] Mandell DJ, Kortemme T (2009) Backbone flexibility in computational protein design. Current opinion in biotechnology 20: 420–428. 19709874 10.1016/j.copbio.2009.07.006

[4] Carlsson J, Coleman RG, Setola V, Irwin JJ, Fan H, et al (2011) Ligand discovery from a dopamine D3 receptor homology model and crystal structure. Nature chemical biology 7: 769–778. 21926995 10.1038/nchembio.662 PMC3197762

[5] Raman S, Vernon R, Thompson J, Tyka M, Sadreyev R, et al (2009) Structure prediction for CASP8 with all-atom refinement using Rosetta. Proteins 77 Suppl 989–99. 19701941 10.1002/prot.22540 PMC3688471

[6] Kinch L, Yong Shi S, Cong Q, Cheng H, Liao Y, et al (2011) CASP9 assessment of free modeling target predictions. Proteins 79 Suppl 1059–73. 21997521 10.1002/prot.23181 PMC3226891

[7] Xu D, Zhang J, Roy A, Zhang Y (2011) Automated protein structure modeling in CASP9 by I-TASSER pipeline combined with QUARK-based ab initio folding and FG-MD-based structure refinement. Proteins 79 Suppl 10147–160. 22069036 10.1002/prot.23111 PMC3228277

[8] DiMaio F, Tyka MD, Baker ML, Chiu W, Baker D (2009) Refinement of protein structures into low-resolution density maps using rosetta. Journal of Molecular Biology 392: 181–190. 19596339 10.1016/j.jmb.2009.07.008 PMC3899897

[9] Raman S, Lange OF, Rossi P, Tyka M, Wang X, et al (2010) NMR structure determination for larger proteins using backbone-only data. Science 327: 1014–1018. 20133520 10.1126/science.1183649 PMC2909653

[10] Schroder GF, Levitt M, Brunger AT (2010) Super-resolution biomolecular crystallography with low-resolution data. Nature 464: 1218–1222. 20376006 10.1038/nature08892 PMC2859093

[11] Raval A, Piana S, Eastwood MP, Dror RO, Shaw DE (2012) Refinement of protein structure homology models via long, all-atom molecular dynamics simulations. Proteins. 10.1002/prot.24098 22513870

[12] Kim DE, Blum B, Bradley P, Baker D (2009) Sampling bottlenecks in de novo protein structure prediction. Journal of Molecular Biology 393: 249–260. 19646450 10.1016/j.jmb.2009.07.063 PMC2760740

[13] Sripakdeevong P, Kladwang W, Das R (2011) An enumerative stepwise ansatz enables atomic-accuracy RNA loop modeling. Proceedings of the National Academy of Sciences of the United States of America 108: 20573–20578. 22143768 10.1073/pnas.1106516108 PMC3251086

[14] Cruz JA, Blanchet MF, Boniecki M, Bujnicki JM, Chen SJ, et al (2012) RNA-Puzzles: a CASP-like evaluation of RNA three-dimensional structure prediction. Rna 18: 610–625. 22361291 10.1261/rna.031054.111 PMC3312550

[15] Chou FC, Sripakdeevong P, Dibrov SM, Hermann T, Das R (2013) Correcting pervasive errors in RNA crystallography through enumerative structure prediction. Nat Methods 10: 74–76. 23202432 10.1038/nmeth.2262 PMC3531565

[16] Sripakdeevong P, Cevec M, Chang AT, Erat MC, Ziegeler M, et al.. (2013) High-resolution structure determination of noncanonical RNA motifs from 1H NMR chemical shifts alone. Nat Methods: under review. 10.1038/nmeth.2876 PMC3985481 24584194

[17] Hammond NB, Tolbert BS, Kierzek R, Turner DH, Kennedy SD (2010) RNA internal loops with tandem AG pairs: the structure of the 5′GAGU/3′UGAG loop can be dramatically different from others, including 5′AAGU/3′UGAA. Biochemistry 49: 5817–5827. 20481618 10.1021/bi100332r PMC2900907

[18] Lyskov S, Chou F-C, Ó Conchúir S, Der BS, Drew K, et al.. (2013) Serverification of Molecular Modeling Applications: the Rosetta Online Server that Includes Everyone (ROSIE). PLOS One: accepted. 10.1371/journal.pone.0063906 PMC3661552 23717507

[19] Grigg JC, Chen Y, Grundy FJ, Henkin TM, Pollack L, et al (2013) T box RNA decodes both the information content and geometry of tRNA to affect gene expression. Proc Natl Acad Sci U S A 110: 7240–7245. 23589841 10.1073/pnas.1222214110 PMC3645572

[20] Zhu K, Pincus DL, Zhao S, Friesner RA (2006) Long loop prediction using the protein local optimization program. Proteins 65: 438–452. 16927380 10.1002/prot.21040

[21] Sellers BD, Zhu K, Zhao S, Friesner RA, Jacobson MP (2008) Toward better refinement of comparative models: predicting loops in inexact environments. Proteins 72: 959–971. 18300241 10.1002/prot.21990 PMC2764870

[22] Mandell DJ, Coutsias EA, Kortemme T (2009) Sub-angstrom accuracy in protein loop reconstruction by robotics-inspired conformational sampling. Nat Methods 6: 551–552. 19644455 10.1038/nmeth0809-551 PMC2847683

[23] Fiser A, Do RK, Sali A (2000) Modeling of loops in protein structures. Protein science: a publication of the Protein Society 9: 1753–1773. 11045621 10.1110/ps.9.9.1753 PMC2144714

[24] Wang C, Bradley P, Baker D (2007) Protein-protein docking with backbone flexibility. J Mol Biol 373: 503–519. 17825317 10.1016/j.jmb.2007.07.050

[25] Harbury PB, Zhang T, Kim PS, Alber T (1993) A switch between two-, three-, and four-stranded coiled coils in GCN4 leucine zipper mutants. Science 262: 1401–1407. 8248779 10.1126/science.8248779

[26] Kuhlman B, Baker D (2000) Native protein sequences are close to optimal for their structures. Proc Natl Acad Sci U S A 97: 10383–10388. 10984534 10.1073/pnas.97.19.10383 PMC27033

[27] Martin AC, Toda K, Stirk HJ, Thornton JM (1995) Long loops in proteins. Protein engineering 8: 1093–1101. 8819975 10.1093/protein/8.11.1093

[28] Leaver-Fay A, Tyka M, Lewis SM, Lange OF, Thompson J, et al (2011) ROSETTA3: an object-oriented software suite for the simulation and design of macromolecules. Methods in enzymology 487: 545–574. 21187238 10.1016/B978-0-12-381270-4.00019-6 PMC4083816

[29] Kortemme T, Kim DE, Baker D (2004) Computational alanine scanning of protein-protein interfaces. Sci STKE 2004: pl2. 14872095 10.1126/stke.2192004pl2

[30] Sontag D, Meltzer R, Globerson A, Jaakkola T, Weiss Y (2008) Tightening LP Relaxations for MAP using Message Passing. CorvallisOregon: AUAI Press. 503–510 p.

[31] Desmet J, De Maeyer M, Hazes B, Lasters I (1992) The dead-end elimination theorem and its use in protein side-chain positioning. Nature 356: 539–542. 21488406 10.1038/356539a0

[32] Eddy SR (2004) What is dynamic programming? Nature biotechnology 22: 909–910. 10.1038/nbt0704-909 15229554

[33] Das R (2011) Four small puzzles that Rosetta doesn't solve. PLoS One 6: e20044. 21625446 10.1371/journal.pone.0020044 PMC3098862

[34] Eswar N, Ramakrishnan C, Srinivasan N (2003) Stranded in isolation: structural role of isolated extended strands in proteins. Protein engineering 16: 331–339. 12826724 10.1093/protein/gzg046

[35] Videau LL, Arendall WB III, Richardson JS (2004) The cis-Pro touch-turn: a rare motif preferred at functional sites. Proteins 56: 298–309. 15211513 10.1002/prot.20101

[36] Chu MH, Liu KL, Wu HY, Yeh KW, Cheng YS (2011) Crystal structure of tarocystatin-papain complex: implications for the inhibition property of group-2 phytocystatins. Planta 234: 243–254. 21416241 10.1007/s00425-011-1398-8 PMC3144364

[37] Lazaridis T, Karplus M (1999) Effective energy function for proteins in solution. Proteins 35: 133–152. 10223287 10.1002/(sici)1097-0134(19990501)35:2<133::aid-prot1>3.0.co;2-n

[38] Rohl CA, Strauss CE, Misura KM, Baker D (2004) Protein structure prediction using Rosetta. Methods Enzymol 383: 66–93. 15063647 10.1016/S0076-6879(04)83004-0

[39] Baird NJ, Zhang J, Hamma T, Ferre-D'Amare AR (2012) YbxF and YlxQ are bacterial homologs of L7Ae and bind K-turns but not K-loops. Rna 18: 759–770. 22355167 10.1261/rna.031518.111 PMC3312563

[40] Hildebrand A, Remmert M, Biegert A, Soding J (2009) Fast and accurate automatic structure prediction with HHpred. Proteins 77 Suppl 9128–132. 19626712 10.1002/prot.22499

[41] Gibson KD, Scheraga HA (1987) Revised algorithms for the build-up procedure for predicting protein conformations by energy minimization. Journal of Computational Chemistry 8: 826–834.

[42] Vajda S, Delisi C (1990) Determining minimum energy conformations of polypeptides by dynamic programming. Biopolymers 29: 1755–1772. 2207285 10.1002/bip.360291408

[43] Hockenmaier J, Joshi AK, Dill KA (2007) Routes are trees: the parsing perspective on protein folding. Proteins 66: 1–15. 17063473 10.1002/prot.21195

[44] Ozkan SB, Wu GA, Chodera JD, Dill KA (2007) Protein folding by zipping and assembly. Proc Natl Acad Sci U S A 104: 11987–11992. 17620603 10.1073/pnas.0703700104 PMC1924571

[45] Chen SJ, Dill KA (2000) RNA folding energy landscapes. Proc Natl Acad Sci U S A 97: 646–651. 10639133 10.1073/pnas.97.2.646 PMC15384

[46] Das R, Andre I, Shen Y, Wu Y, Lemak A, et al (2009) Simultaneous prediction of protein folding and docking at high resolution. Proc Natl Acad Sci U S A 106: 18978–18983. 19864631 10.1073/pnas.0904407106 PMC2770007

[47] Vallurupalli P, Bouvignies G, Kay LE (2012) Studying “invisible” excited protein states in slow exchange with a major state conformation. J Am Chem Soc 134: 8148–8161. 22554188 10.1021/ja3001419

[48] Ollikainen N, Smith CA, Fraser JS, Kortemme T (2013) Flexible backbone sampling methods to model and design protein alternative conformations. Methods Enzymol 523: 61–85. 23422426 10.1016/B978-0-12-394292-0.00004-7 PMC3750959

[49] Adams PD, Baker D, Brunger AT, Das R, Dimaio F, et al.. (2013) Advances, Interactions, and Future Developments in the CNS, Phenix, and Rosetta Structural Biology Software Systems. Annu Rev Biophys. 10.1146/annurev-biophys-083012-130253 PMC8262117 23451892

[50] Nocedal J, Wright SJ (2006) Numerical Optimization: Springer.

[51] Gray JJ, Moughon S, Wang C, Schueler-Furman O, Kuhlman B, et al (2003) Protein-protein docking with simultaneous optimization of rigid-body displacement and side-chain conformations. Journal of Molecular Biology 331: 281–299. 12875852 10.1016/s0022-2836(03)00670-3

[52] Kortemme T, Morozov AV, Baker D (2003) An orientation-dependent hydrogen bonding potential improves prediction of specificity and structure for proteins and protein-protein complexes. Journal of Molecular Biology 326: 1239–1259. 12589766 10.1016/s0022-2836(03)00021-4

[53] Canutescu AA, Dunbrack RL Jr (2003) Cyclic coordinate descent: A robotics algorithm for protein loop closure. Protein Sci 12: 963–972. 12717019 10.1110/ps.0242703 PMC2323867

[54] Vernon R (2010) Structure Prediction with Experimental Constraints. Seattle: University of Washington.

[55] Word JM, Lovell SC, Richardson JS, Richardson DC (1999) Asparagine and glutamine: using hydrogen atom contacts in the choice of side-chain amide orientation. Journal of Molecular Biology 285: 1735–1747. 9917408 10.1006/jmbi.1998.2401

[56] Sripakdeevong P, Kladwang W, Das R (2011) An enumerative stepwise ansatz enables atomic-accuracy RNA loop modeling. Proc Natl Acad Sci USA: in press. 10.1073/pnas.1106516108 PMC3251086 22143768

[57] Condor High Throughput Computing. DAGMan (Directed Acyclic Graph Manager).

[58] Schwede T, Sali A, Honig B, Levitt M, Berman HM, et al (2009) Outcome of a workshop on applications of protein models in biomedical research. Structure 17: 151–159. 19217386 10.1016/j.str.2008.12.014 PMC2739730

[59] Kratzner R, Debreczeni JE, Pape T, Schneider TR, Wentzel A, et al (2005) Structure of Ecballium elaterium trypsin inhibitor II (EETI-II): a rigid molecular scaffold. Acta crystallographica Section D, Biological crystallography 61: 1255–1262. 16131759 10.1107/S0907444905021207

[60] McDonald IK, Thornton JM (1994) Satisfying hydrogen bonding potential in proteins. J Mol Biol 238: 777–793. 8182748 10.1006/jmbi.1994.1334

[61] Harris GW, Pickersgill RW, Howlin B, Moss DS (1992) The segmented anisotropic refinement of monoclinic papain by the application of the rigid-body TLS model and comparison to bovine ribonuclease A. Acta crystallographica Section B, Structural science 48 (Pt. 1): 67–75. 10.1107/s0108768191006663 1616693

[62] Buckle AM, Henrick K, Fersht AR (1993) Crystal structural analysis of mutations in the hydrophobic cores of barnase. Journal of Molecular Biology 234: 847–860. 8254677 10.1006/jmbi.1993.1630

[63] Urakubo Y, Ikura T, Ito N (2008) Crystal structural analysis of protein-protein interactions drastically destabilized by a single mutation. Protein science: a publication of the Protein Society 17: 1055–1065. 18441234 10.1110/ps.073322508 PMC2386745

[64] McPhalen CA, Svendsen I, Jonassen I, James MN (1985) Crystal and molecular structure of chymotrypsin inhibitor 2 from barley seeds in complex with subtilisin Novo. Proceedings of the National Academy of Sciences of the United States of America 82: 7242–7246. 16593622 10.1073/pnas.82.21.7242 PMC390825

[65] McPhalen CA, James MN (1987) Crystal and molecular structure of the serine proteinase inhibitor CI-2 from barley seeds. Biochemistry 26: 261–269. 3828302 10.1021/bi00375a036

[66] Savva R, McAuley-Hecht K, Brown T, Pearl L (1995) The structural basis of specific base-excision repair by uracil-DNA glycosylase. Nature 373: 487–493. 7845459 10.1038/373487a0

