# Supplementary material for: Atomic-Accuracy Prediction of Protein Loop Structures through an RNA-Inspired Ansatz
Source: PLoS One. 2013 Oct 21;8(10):e74830. doi: 10.1371/journal.pone.0074830 (PMC3804535; doi:10.1371/journal.pone.0074830)
Supplement: Table S1 — Comparison of all loop modeling methods in 20-residue PLOP/Rosetta benchmark. (PDF) [file pone.0074830.s004.pdf]

Supporting Information Table S1. Comparison of all loop modeling methods in 20-residue PLOP/Rosetta benchmark.

Target	KIC, Number of models[a]	Cα RMSD to crystallographic loop (Å)						
		KIC, lowest RMSD	KIC, best of 5 (rank)	Comparison of lowest energy models				
				KIC	KIC[b]	Frag.Assemb.[b]	PLOP[c]	SWA
1a8d	4906	0.71	0.71 (1)	0.71	6.9	5.4	2.8	0.42
1arb	6635	0.59	1.54 (1)	1.54	1.0	1.6	2.6	0.48
1bhe	7645	0.50	0.61 (1)	0.61	0.8	7.1	0.7	0.30
1bn8	6193	0.51	0.92 (1)	0.92	0.7	2.5	2.6	1.27
1c5e	12198	0.34	0.36 (1)	0.36	0.5	0.8	1.7	1.25
1cb0	9302	0.46	0.56 (1)	0.56	0.6	1.0	0.3	0.64
1cnv	6879	0.84	1.59 (1)	1.59	1.4	2.3	3.3	1.59
1cs6	7513	0.93	1.94 (4)	2.87	3.0	2.5	3.5	0.79
1dqz	8525	0.52	0.76 (1)	0.76	0.7	1.9	0.6	0.48
1exm	6019	0.65	0.98 (1)	0.98	0.9	0.6	0.5	0.62
1f46	14228	0.50	0.57 (1)	0.57	2.5	2.1	1.1	0.38
1i7p	7119	0.39	0.49 (2)	2.83	2.7	0.7	0.3	1.61
1m3s[d]	11730	0.27	0.36 (1)	0.36	6.3	3.6	5.6	3.24
1ms9	4712	0.24	0.39 (1)	0.39	0.4	2.5	2.5	0.34
1my7	9451	0.35	0.75 (1)	0.75	2.3	2.0	0.9	0.51
1oth	8567	0.31	0.39 (1)	0.39	0.6	0.6	0.7	0.71
1oyc	16878	1.68	3.03 (2)	4.53	4.0	3.2	1.2	0.39
1qlw	5860	1.00	1.24 (1)	1.24	1.0	3.3	1.4	4.98
1t1d	10378	0.45	0.90 (1)	0.90	0.8	0.5	1.0	0.41
2pia	6479	0.67	1.10 (1)	1.10	1.0	1.1	0.5	1.06
Mean	8053	0.60	0.91 (1)	1.15	1.9	2.3	1.7	**1.07**
Median	7316	0.51	0.76 (1)	0.83	1.0	2.1	1.2	**0.63**
RMSD < 1.0 Å		18	14	13	10	6	8	**14**

[a] Results from applying 6400 CPU-hours of computation (Intel Xeon E5345 2.33 GHz). For 1OYC case, 16000 CPU-hours were expended.
[b] From Supplementary Table 2 in [Mandell, Coutsias, and Kortemme (2009), *Nat. Methods* 6:551-552] Number of KIC models in previous study was 1000.
[c] From Supplementary Table S4 ("new protocol") in [Sellers et al. (2008), *Proteins* 72:959-971].
[d] SWA and repeated KIC runs included two crystallographic neighbors that interact with loop.
