# Supplementary material for: Atomic-Accuracy Prediction of Protein Loop Structures through an RNA-Inspired Ansatz
Source: PLoS One. 2013 Oct 21;8(10):e74830. doi: 10.1371/journal.pone.0074830 (PMC3804535; doi:10.1371/journal.pone.0074830)
Supplement: Table S2 — Energy comparisons to determine convergence and conformational sampling efficiency. (PDF) [file pone.0074830.s005.pdf]

Supporting Information Table S2. Energy comparisons to estimate convergence and conformational sampling efficiency.

Target	Cα RMSD Best of 5 (rank)	All–atom Rosetta Energy differences[c]		
		SWA: (2) – (1)[d]	SWA –NATIVE	SWA – KIC
1a8d[a]	0.42 (1)	8.8	–0.4	–4.3
1arb[a]	0.48 (1)	5.5	–0.2	–1.6
1bhe	0.30 (1)	2.9	2.2	–5.5
1bn8	0.63 (2)	0.8	–1.2	–2.0
1c5e	0.44 (2)	0.3	0.4	1.1
1cb0[a]	0.64 (1)	8.4	0.5	–0.6
1cnv	1.59 (1)	5.7	0.0	–5.5
1cs6[a]	0.79 (1)	0.7	0.8	–2.1
1dqz[a]	0.48 (1)	3.7	1.8	–0.0
1exm[a]	0.62 (1)	4.9	2.4	0.3
1f46	0.38 (1)	4.0	–3.0	–1.1
1i7p	0.43 (3)	1.5	0.0	–4.4
1m3s[a,g]	0.27 (2)	1.6	0.5	4.5
1ms9	0.34 (1)	0.5	–0.4	–0.2
1my7	0.51 (1)	0.7	0.3	1.5
1oth[a]	0.71 (1)	3.5	1.4	–1.5
1oyc	0.39 (1)	4.4	1.4	–2.6
1qlw	0.66 (3)	0.4	3.0	–0.1
1t1d	0.41 (1)	0.6	0.5	0.4
2pia[a]	0.83 (3)	7.0	–0.1	–1.8
1alc[a]	0.58 (2)	3.1	0.3	-8.1
1msp[a]	0.73 (1)	7.0	0.3	11.6
1w7z	0.79 (1)	1.8	–0.1	-9
2tgi	0.53 (3)	1.6	–3.4	-12
1ppn	0.89 (1)	1.0	–1.3	-9.1
1bni	1.10 (4)	1.4	2.9	1.4
2ci2	3.44 (5)	0.0	–0.9	4
1udg	2.43 (3)	1.7	0.8	3.3
1arp[a]	1.60 (1)	0.5	–0.7	-11.5
1huw[a]	1.35 (1)	4.2	1.7	-11.8
1rhd[a]	0.82 (2)	0.8	4.5	4.1
7cat[a]	7.69 (1)	0.1	7.5	6.1
1thg[a]	0.80 (1)	4.1	12.3	–26.2
1c5e[a,b]	0.41 (1)	1.8	1.4	–14.8
1rhd[a,b]	12.20 (5)	0.5	14.2	-0.5
testA	0.91 (3)	0.4	–1.7	0.3
testB	0.91 (1)	0.8	–5.7	-6.1
testC	0.54 (1)	1.3	–5.1	-0.9
testD	0.52 (1)	2.7	–1.5	-0.8
3v7e[a]	0.53 (1)	3.7	n.d.[e]	n.d.[e]
Mean	1.27 (1)	2.4	0.9	–2.7[f]
Median	0.64 (1)	1.6	0.3	–1.1[f]

[a] SWA runs carried out with simplified O(N) calculation scheme; see Supporting Information methods.
[b] Longer and shorter variants of loops were modeled separately; see Supporting Information Table S1.
[c] Energies given in Rosetta units (approximately 1 k_BT).
[d] Energy difference between second lowest energy SWA model and lowest energy SWA model, used to assess convergence.
[e] For 3v7e (blind test, RNA-binding protein ybxF), optimized crystallographic energies cannot be compared to loops built on comparative model due to differences in starting scaffold.
[g] Test included two crystallographic neighbors that interact with loop.
